# Albatredines A and B, a pair of epimers with unusual natural heterocyclic skeletons from edible mushroom *Albatrellus confluens*†

**DOI:** 10.1039/c8ra04226h

**Published:** 2018-07-02

**Authors:** Shuaibing Zhang, Ying Huang, Shijun He, Heping Chen, Zhenghui Li, Bin Wu, Jianping Zuo, Tao Feng, Jikai Liu

**Affiliations:** School of Pharmaceutical Sciences, South-Central University for Nationalities Wuhan 430074 China tfeng@mail.scuec.edu.cn jkliu@mail.kib.ac.cn; Leibniz Research Group – Biobricks of Microbial Natural Product Syntheses, Leibniz Institute for Natural Product Research and Infection Biology (HKI) Adolf–Reichwein–Str. 23 07745 Jena Germany; State Key Laboratory of Drug Research, Shanghai Institute of Materia Medica, Chinese Academy of Sciences Shanghai 201203 China jpzuo@simm.ac.cn

## Abstract

A chemical study of the common species *Albatrellus confluens* present in Yunnan province, southwest China led to the identification of a pair of epimers named albatredines A (1) and B (2). They feature a natural unprecedented 1,2,4-oxadiazolidin-5-one skeleton. The acyl substitution pattern and complete configurational assignments were deduced from the comparison between experimental and theoretical ^13^C NMR and ECD data, respectively. Bioassay results showed that compound 1 exhibited a weak immunosuppressive activity against the concanavalin A-induced T lymphocyte cell proliferation (IC_50_ 2.99 μM).

## Abstract 2

A chemical study of the common species *Albatrellus confluens* present in Yunnan province, southwest China led to the identification of a pair of epimers named albatredines A (1) and B (2).

## Graphical abstract

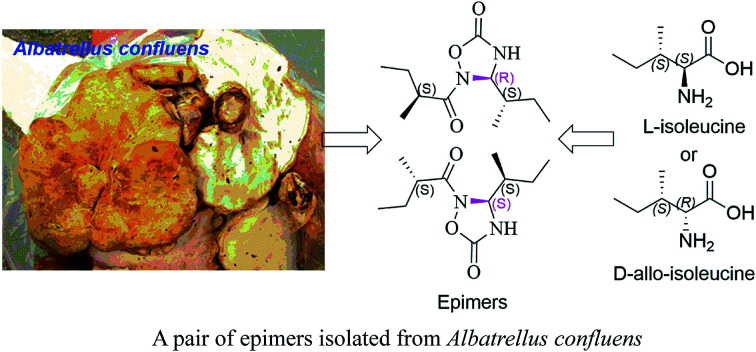

## Introduction

Nitrogen-containing heterocyclic scaffolds are basic and prevalent structural units of many drugs, such as Sovaldi, Abilify, and Nexium.^1^ Nitrogen-containing compounds originating from mushrooms are a large group of diverse secondary metabolites^2,3^ (characterized by various chiral structures with different bioactivities), such as enantiomers and epimers. These metabolites are not equally effective in blocking the effect of some receptors and may have a large difference in their ability to cure some diseases, the reason for which is still not known. For instance, in the formation of the peptide for the origin of life, l-amino acids were chosen instead of d-amino acids and d-sugars were selected instead of l-sugars, which was an amazing natural choice.^4^ However, non-crystallized natural products with multiple chiral centers, particularly the lower molecular weight compounds with more heteroatoms, dwarf the determination of their planar structures and absolute configurations to a large extent.

**Fig. 1:**
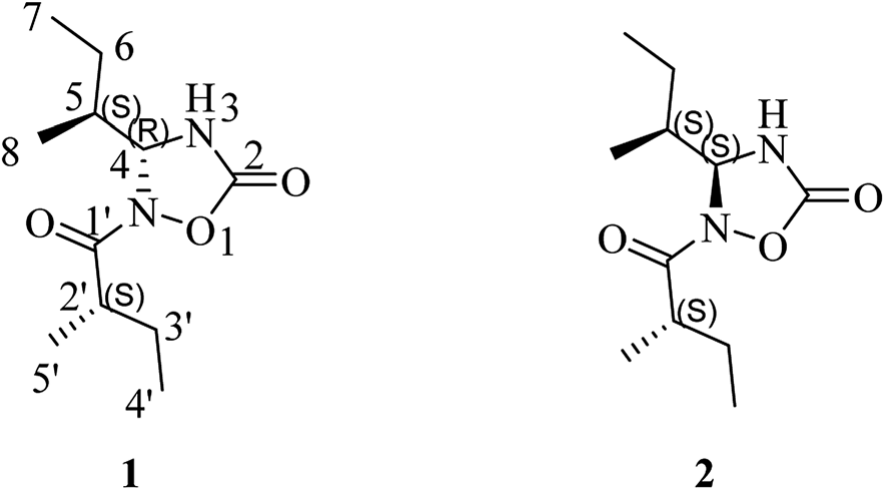
Structures of albatredines A (1) and B (2).

The edible mushroom *Albatrellus confluens*, which belongs to the family Albatrellaceae, is widely distributed in China. Although the phytochemical investigations on *A. confluens* have been extensively reported,^5–12^ an interesting discovery has been made in the lower-polarity fraction of the extractions from the fruiting bodies of *A. confluens* collected in southwest China, Yunnan, which led to the isolation and structural elucidation of a pair of undescribed nitrogen-containing heterocyclic compounds albatredines A (1) and B (2) (Fig. 1). Structurally, albatredines A and B are quite unprecedented in nature, possessing five-membered 1,2,4-oxadiazolidin-5-one heterocyclic skeletons. Although the efforts to cultivate single crystals of these compounds have failed, the absolute configurations of these compounds were unambiguously determined by ECD calculations as well as ^13^C NMR calculations.^13^ Bioassay results indicated that compound 1 exhibited inhibitory activity against concanavalin A-induced T lymphocyte cell proliferation, an important target in the treatment of immunosuppression.

## Results and discussion

**Fig. 2:**
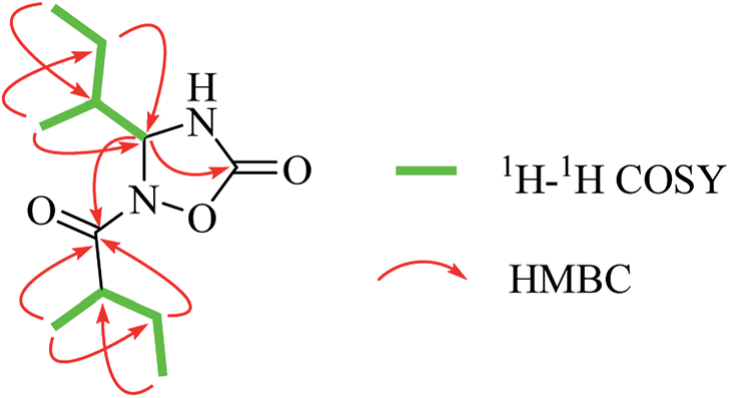
^1^H–^1^H COSY and key HMBC correlations of 1.

(+)-HRESIMS analysis of 1 revealed a major ion peak at *m/z* 229.1544, which was consistent with the molecular formula C_11_H_21_N_2_O_3_ [M + H]^+^. First inspection of the ^1^H NMR spectrum (Table 1) evidenced two 2-methylbutyl units with methyl signals at *δ*_H_ 0.92 (t, 3H, *J* = 7.5 Hz, H-7), 0.98 (d, 3H, *J* = 6.8 Hz, H-8), 0.95 (t, *J* = 7.5 Hz, 3H, H-4′) and 1.08 (d, *J* = 6.9 Hz, 3H, H-5′), which was also supported by the ^1^H–^1^H COSY correlations of H-4/H-5/H-6/H-7 and H-5/H-8 together with H-5′/H-2′/H-3′/H-4′ as well as the HMBC correlations of H-8/C-4 and C-6; H-7/C-5, H-6/C-8 and C-4; H-4′/C-2′, H-3′/C-5′, and C-1′; and H-5′/C-1′. The critical HMBC correlations from the proton at *δ*_H_ 5.55 (d, 1H, *J* = 4.4 Hz, H-4) to C-2 and C-1′ suggested that the two carbonyl moieties (*δ*_C_ 157.1; 182.5) were located at the *meta* position of C-4 (Fig. 2). Because no HMBC was observed from the H–N signal, we compared the experimental and theoretical ^13^C NMR chemical shifts of all possible substitution patterns. Choosing the location of the 2-methylbutyryl on one primary amine in five-membered heterocycles afforded two possible substitutions, and forming a four-membered heterocycle afforded one possible substitution. Among the three possible substitution patterns, the most probable locations of the acyls are shown in Fig. 1 with 99.92% confidence (Table S20†).^14–17^ Accordingly, the planar structure of 1 was determined to be a new natural N-containing derivative bearing a 1,2,4-oxadiazolidin-5-one group.

**Table 1:** ^13^C NMR (150 MHz) and ^1^H NMR (600 MHz) spectroscopic data of Albatredine A (1) (*δ* in ppm)

No.	*δ*_C_ a	*δ*_H_ a	*δ*_C_ b	*δ*_H_ b
2	157.12		154.64	
4	76.58	5.55(d, 4.4)	74.67	5.46(dd, 4.7,1.9)
5	41.77	1.70(m)	39.8	1.59(m)
6	24.95	1.53(m), 1.16(m)	23.36	1.45(m), 1.08(m)
7	11.71	0.92(t, 7.4)	11.22	0.91(t, 7.3)
8	13.33	0.98(d, 6.8)	12.89	0.87(d, 6.8)
1′	182.45		180.22	
2′	39.64	2.87(m)	37.63	2.78(m)
3′	28.02	1.66(m), 1.53(m)	26.39	1.59(m), 1.48(m)
4′	11.69	0.95(t, 7.4)	11.16	0.85(t, 7.4)
5′	15.79	1.08(d, 6.9)	15.26	1.01(d, 6.9)
–NH				9.16(s)

a Measured in methanol-*d*_4_.

b Measured in DMSO-*d*_6_.

**Fig. 3:**
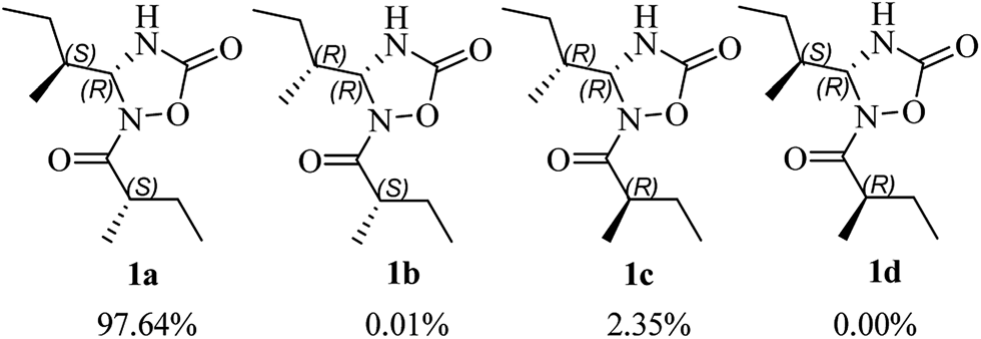
DP4+ probabilities of ^13^C and ^1^H NMR data for the diastereoisomers of 1.

**Fig. 4:**
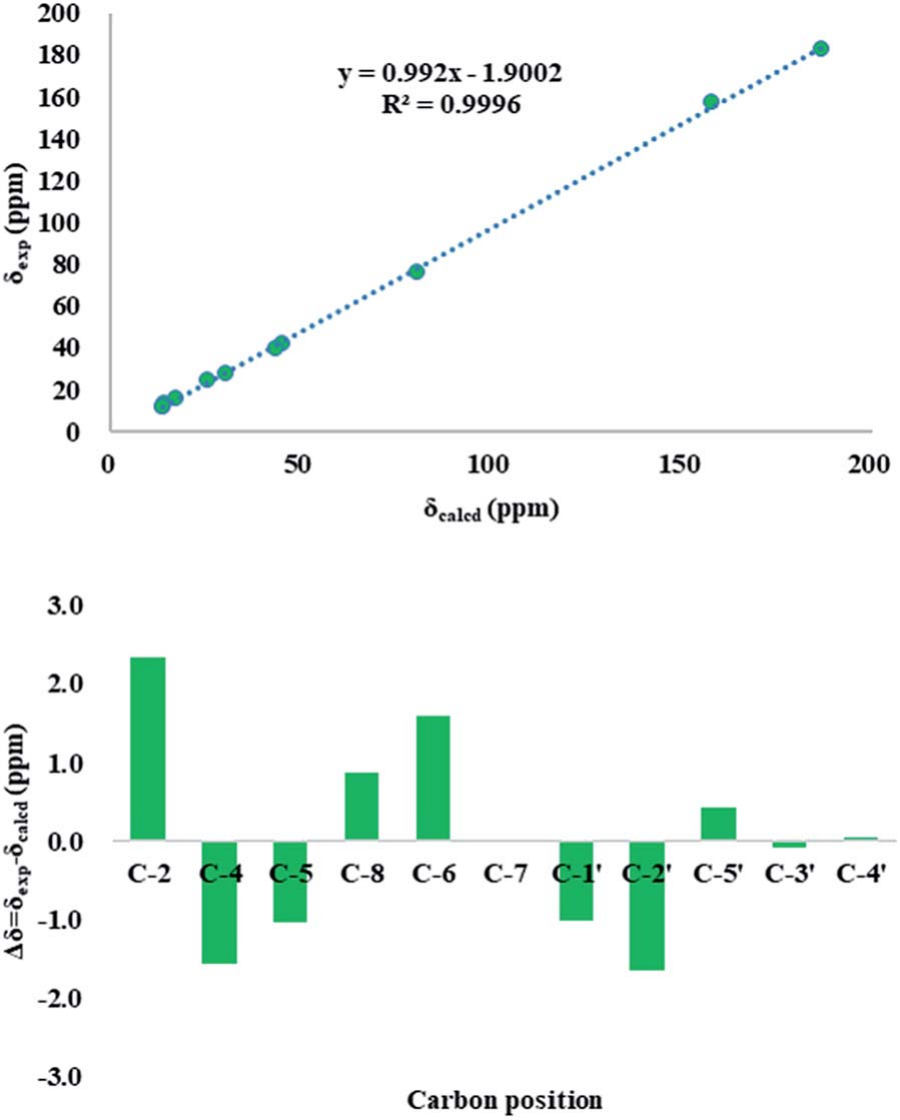
Up: regression analysis of experimental *versus* calculated ^13^C NMR chemical shifts of 1 at the mPW1PW91/6-311g(d,p) level; linear fitting is shown as a line; down: relative chemical shift errors between scaled ^13^C NMR and experimental ^13^C NMR for 1 (*δ*_corr_ obtained by the linear fit of *δ*_exp_*versus δ*_calcd_).

The use of the DP4+ probability was also required to determine the relative configuration of 1. On the basis of the relative configuration, four stereoisomers, (4*R*,5*S*,2′*S*)-1 (1a), (4*R*,5*R*,2′*S*)-1 (1b), (4*R*,5*R*,2′*R*)-1 (1c), (4*R*,5*S*,2′*R*)-1 (1d) were observed. As shown in Fig. 3, the relative configuration was established as 4R*,5S*,2′S* with 97.64% confidence (Table S13†). The results also revealed that the correlation coefficient (*R*^2^) between calculated and experimental data from the linear regression analysis was 0.9996, and the root-mean-square deviation (RMSD) was 1.21 ppm, which further provided solid evidence for the structural rationality of 1 (Fig. 4).

**Fig. 5:**
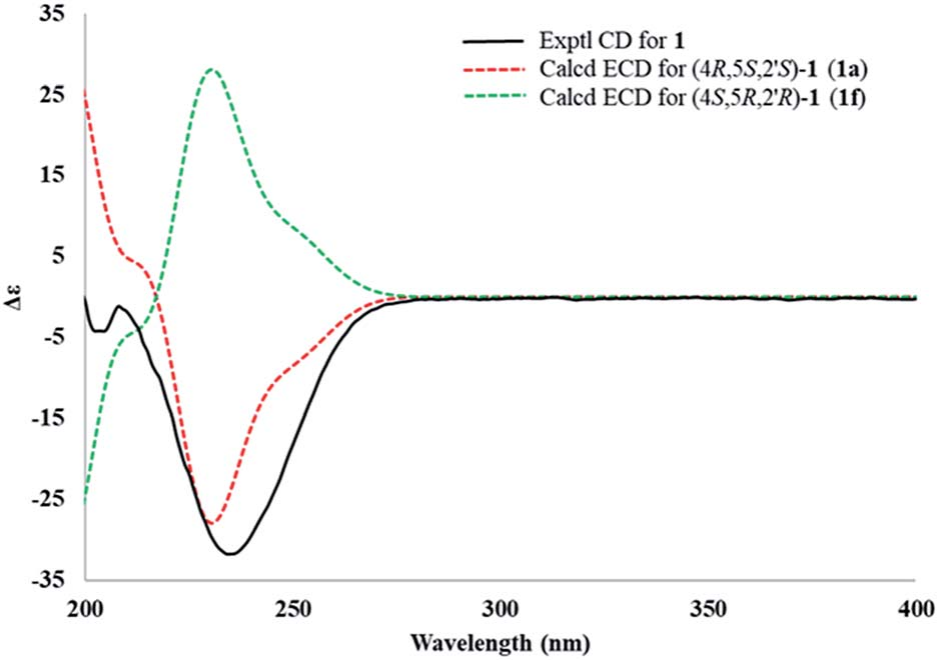
Comparison between the experimental (line) and theoretical (dashed) ECD spectra of the two diastereoisomers of 1.

Electronic circular dichroism (ECD) calculations were used to determine the absolute configuration of 1 by the time dependent density functional theory (TDDFT) method at the CAM-B3LYP/6-311+G (d,p) level in gas phase. Two stereoisomers, (4*R*,5*S*,2′*S*)-1 (1a) and (4*S*,5*R*,2′*R*)-1 (1f), exist on the basis of the relative configuration. Comparison of theoretically calculated and experimental ECD curves (Fig. 5) showed that the calculated curves of 1a were similar to the experimental data, indicating the absolute configuration of 1 as 4*R*,5*S*,2′*S*.

Compound 2 was isolated as a white powder. Its molecular formula was determined as C_11_H_21_N_2_O_3_ by HRESIMS, which was the same as that of 1. The NMR data (Tables 1 and 2), UV and IR spectra of 2 and 1 had remarkable resemblances. In addition, the experimental ECD spectra of 1 and 2 also possessed mirror symmetry. All the abovementioned results illustrated that 1 and 2 were a pair of epimers. Correspondingly, calculated ECD (Fig. S27†) and DP4+ probabilities (Table S13†) were also used to tackle the absolute configurations, which were confirmed as 4*S*,5*S*,2′*S*.

**Table 2:** ^13^C NMR (150 MHz) and ^1^H NMR (600 MHz) spectroscopic data of Albatredine B (2) (*δ* in ppm)

No.	*δ*_C_ a	*δ*_H_ a
2	156.96	
4	76.78	5.55(d, 4.7)
5	41.44	1.78–1.69(m)
6	24.97	1.51(m), 1.20(m)
7	12.06	0.97(t, 7.5)
8	13.12	0.95(d, 6.8)
1′	182.2	
2′	39.58	2.92–2.82(m)
3′	27.02	1.65(m), 1.46(m)
4′	11.84	0.91(t, 7.5)
5′	17.65	1.18(d, 6.9)

a Measured in methanol-*d*_4_.

**Scheme 1:**
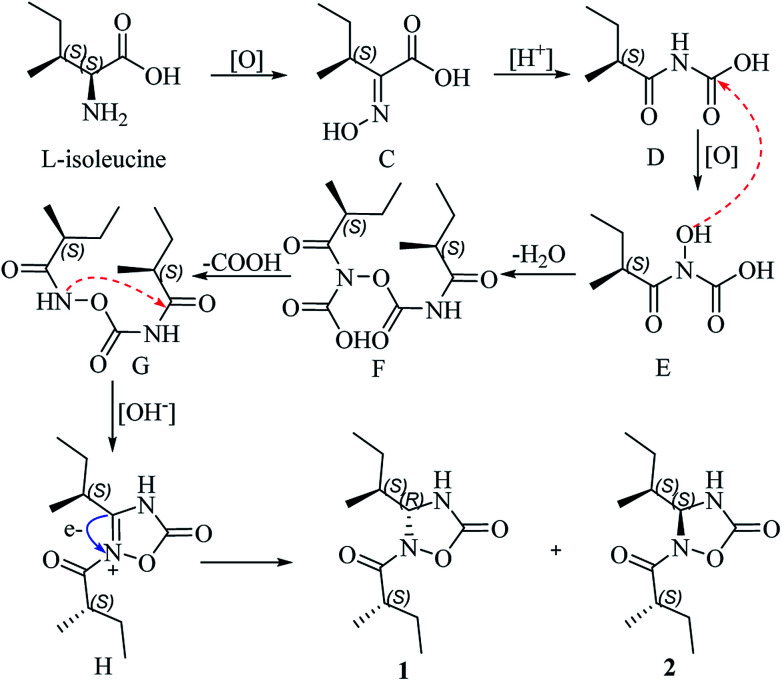
Proposed biosynthetic pathways for 1 and 2.

Albatredines A and B represent nor-peptides with unprecedented natural heterocyclic skeletons, which aroused our interest in their plausible biogenesis. Biosynthetically, this pair of epimers might possibly be traced back to l-isoleucine since they possessed this common side chain. The probable biogenetic pathways for 1 and 2 are proposed (Scheme 1). l-isoleucine was oxygenated at the amino site to give the key (*S*,*E*)-2-(hydroxyimino)-3-methylpentanoic acid (C), which could induce a rearrangement reaction under acidic condition to afford D. Furthermore, the generation of D was also followed by an oxidation at the amino site to generate E, which proceeded with D to form the intermediate F involving the elimination of water. Correspondingly, a decarboxylative reaction of F took place to produce G, which could trigger an intramolecular SN_2_ nucleophilic addition in basic condition to yield the intermediate H. The intermediate H further underwent an electron transfer to give a pair of epimers at C-4.

Immunosuppressants are an important class of clinical drugs and an essential prerequisite for successful organ transplantation and treatment of various autoimmune diseases, such as psoriasis, pemphigus, myasthenia gravis, multiple sclerosis, systemic lupus and rheumatoid arthritis.^18^ Thus, we have evaluated the immunosuppressive activity of 1 and 2 by a previously reported method.^19^ In the experiment, cyclosporin A (CsA) was used as the positive control. Compound 1 exhibited weak activity against concanavalin A-induced T lymphocyte cell proliferation with an IC_50_ value of 2.99 μM (Table 3). However, compound 2 did not show any activity, which could explain that there is a difference between the epimers to some extent.

**Table 3:** Immunosuppressive tests of 1 and 2

No.	CC_50_ (μM)	ConA-induced T-cell proliferation		LPS-induced B-cell proliferation	
		IC_50_ (μM)	SI^a^	IC_50_ (μM)	SI^a^
1	21.05	2.99	7.04	11.46	1.84
2	>160	>200	-	>200	-
CsA	4.63	0.02	189.43	0.77	5.99

a SI was determined as the ratio of the concentration of the compound that reduced the cell viability to 50% (CC_50_) to the concentration of the compound needed to inhibit the proliferation by 50% relative to the control value (IC_50_). “-” stands for inactive.

## Conclusions

In summary, chemical investigations on the famous edible mushroom *A. confluens* were conducted, which resulted in the isolation of a pair of epimers, albatredines A (1) and B (2), bearing a previously undescribed natural heterocyclic scaffold. Their structures were unambiguously determined through extensive spectroscopic analysis in combination with computational approaches. Moreover, immunosuppressive bioassay results suggested that the discovery of new types of heterocyclic compounds such as these from natural sources provides new possibilities to explore new immunosuppressants by the inhibition of T cell proliferation.

## Experimental section

### General experimental procedures

Optical rotations (OR) were recorded on a JASCO P-1020 digital polarimeter (Horiba, Kyoto, Japan). UV/Vis spectra were obtained using a Shimadzu UV2401PC spectrometer (Shimadzu, Kyoto, Japan). CD spectra were obtained on an Applied Photophysics Chirascan Circular Dichroism Spectrometer (Applied Photophysics Limited, Leatherhead, Surrey, UK). IR spectra were obtained using a Bruker Tensor 27 FT-IR spectrometer (Bruker Optics, Inc., Billerica, MA) with KBr pellets. HR ESI-MS spectra were recorded on an Agilent 6200 Q-TOF MS system (Agilent Technologies, Santa Clara, CA, USA). NMR spectra were obtained on a Bruker Avance III 600 MHz spectrometer (Bruker BioSpin GmbH, Karlsruhe, Germany). Silica gel (200–300 mesh, Qingdao Haiyang Chemical Co., Ltd., P. R. China) and Sephadex LH-20 (Amersham Biosciences, Sweden) were used for column chromatography (CC). Medium-pressure liquid chromatography (MPLC) was performed on a Büchi Sepacore System equipped with pump manager C-615, pump modules C-605 and a fraction collector C-660 (Büchi Labortechnik AG, Switzerland), and columns packed with Chromatorex C-18 (40–75 μm, Fuji Silysia Chemical Ltd., Japan). Preparative HPLC was performed on an Agilent 1260 liquid chromatography system equipped with two types of Zorbax SB-C18 columns (9.4 mm × 150 mm and 21.2 mm × 150 mm, particle size 5 μm). Chiral phase HPLC analysis/preparation was performed on an Agilent 1100 liquid chromatography system equipped with a Diacel CHIRAL-PAK AS-H column (particle size 5 μm, dimensions 4.6 mm × 250 mm).

#### Fungal material

The fresh fruiting bodies of *Albatrellus confluens* were obtained from the Tiger Leaping Gorge in Yunnan province, China, in August 2012 and identified by Mr Li Zhenghui, South-Central University for Nationalities. A voucher specimen (No. 20120815A) was deposited at the herbarium of Kunming Institute of Botany, the Chinese Academy of Sciences.

#### Extraction and isolation

The air-dried and powdered fruiting bodies of *A. confluens* (2.5 kg) were soaked in 95% ethanol. The extract was evaporated under reduced pressure and partitioned between ethyl acetate and water four times to obtain an ethyl acetate crude extract (25.0 g). The crude extract was subject to normal CC with a stepwise gradient of CHCl_3_/MeOH (v/v 100 : 0–1 : 1) to afford sixteen fractions (A–P). Fraction C (2.2 g) was subjected to MPLC with a gradient solvent system of MeOH/H_2_O (v/v 20 : 80–100 : 0, 25 mL min^−1^) to obtain 20 subfractions (C1–C20). Subfraction C9 (150 mg) was further purified by Sephadex LH-20 (MeOH) to give 12 subfractions (C9-1 to C9-12). Generation of C9-5 was followed by prep-HPLC to yield compounds 1 (4.4 mg, MeCN–H_2_O: 28%, 20 min, 10 mL min^−1^) and 2 (4.9 mg, MeCN–H_2_O: 30%, 18 min, 10 mL min^−1^).

##### Albatredine A (1)

White powder; [*α*]^20^_D_ − 179.6 (*c* 0.10 MeOH); UV (MeOH) *λ*_max_ (log *ε*) 208 nm (3.91) CD (MeOH) *λ* (Δ*ε*) 235 (−39.6); IR (KBr) *ν*_max_ 3290, 2969, 1785, 1701, 1462, 1203, 937 cm^−1^; HRESI(+)MS *m*/*z* 229.1544 [M + H]^+^ (calcd. for C_11_H_20_N_2_O_3_H, 229.1547).

##### Albatredine B (2)

White powder; [*α*]^20^_D_ + 56.3 (*c* 0.10 MeOH); UV (MeOH) *λ*_max_ (log *ε*) 207 nm (3.20); CD (MeOH) *λ* (Δ*ε*) 234 (+31.8); IR (KBr) *ν*_max_ 3286, 2968, 1786, 1700, 1462, 1203, 937 cm^−1^; HRESI(+)MS *m*/*z* 229.1547 [M + H]^+^ (calcd. for C_11_H_20_N_2_O_3_H, 229.1547).

### Immunosuppressive activities assay

#### Live subject statement

The animal experiment was carried out in strict accordance with the institutional ethical guidelines on animal care and was approved by the Institute Animal Care and Use Committee (IACUC) at the Shanghai Institute of Materia Medica, Chinese Academy of Sciences (IACUC protocol# 2017-03-ZJP-62).

#### Preparation of spleen cells from mice

Female BALB/c mice were sacrificed by cervical dislocation, and the spleens were removed aseptically. Mononuclear cell suspensions were prepared after the cell debris and clumps were removed. Erythrocytes were depleted with an ammonium chloride buffer solution. Lymphocytes were washed and resuspended in the RPMI 1640 medium supplemented with 10% FBS, penicillin (100 U mL^−1^), and streptomycin (100 mg mL^−1^).

#### Cytotoxicity assay

Cytotoxicity was assessed with a Cell Counting Kit-8 (CCK-8) assay. Briefly, fresh spleen cells were obtained from female BALB/c mice (Bagg albino) (18–20 g). Spleen cells (1 × 10^6^ cells) were cultured at 37 °C for 48 h in 96-well flat plates in the presence or absence of various concentrations of compounds in a humidified and 5% CO_2_-containing incubator. A certain amount of CCK-8 was added to each well at the final 8-10 h of culture. Towards the end of the culture, we measured the OD values with a microplate reader (Bio Rad 650) at 450 nm. The cytotoxicity of each compound was expressed as the concentration of the compound that reduced the cell viability to 50% (CC50).

#### T cell and B cell function assay

Fresh spleen cells were obtained from female BALB/c mice (18–20 g). The 5 × 10^5^ spleen cells were cultured at the same conditions as those mentioned above. The cultures, in the presence or the absence of various concentrations of compounds, were stimulated with 5 μg mL^−1^ of concanavalin A (ConA) or 10 μg mL^−1^ of LPS (lipopolysaccharide) to induce T cells' or B cells' proliferative responses, respectively. Proliferation was assessed in terms of uptake of [^3^H]-thymidine during 8 h of pulsing with 25 μL per well of [^3^H]-thymidine and then, the cells were harvested onto glass fiber filters. The incorporated radioactivity was counted using a Beta scintillation counter (MicroBeta Trilux, PerkinElmer Life Sciences). The immunosuppressive activity of each compound was expressed as the concentration of the compound that inhibited the ConA-induced T cell proliferation or LPS-induced B cell proliferation to 50% (IC_50_) of the control value.

### Computational methods

#### NMR calculation

The NMR calculations for compounds were performed using Gaussian 09. Conformation searches based on molecular mechanics with the MMFF94s force field were performed for (4*R*,5*S*,2′*S*)-1 (1a), (4*R*,5*R*,2′*S*)-1 (1b), (4*R*,5*R*,2′*R*)-1 (1c) and (4*R*,5*S*,2′*R*)-1 (1d), which had 15, 15, 16 and 15 conformers with populations higher than 1%, respectively.^20,21^ All these conformers were further optimized by the density functional theory method at the B3LYP/6-31G(d) level in Gaussian 09 program package,^22^ which led to eleven (1a1 to 1a11), twelve (1b1 to 1b12), eleven (1c1 to 1c11) and ten (1d1 to 1d10) conformers within a 2.0 kcal mol^−1^ energy threshold from global minimum, respectively. These predominant conformers were subjected to NMR calculation. Gauge-Independent Atomic Orbital (GIAO) calculations of ^13^C NMR of the conformers were accomplished by the density functional theory (DFT) at the mPW1PW91/6-311g(d,p) level in methanol with the PCM model. The ^13^C NMR chemical shift of TMS was calculated in the same level and used as the reference. The calculated NMR data of these conformers were averaged according to the Boltzmann distribution theory and their relative Gibbs free energies.^13^ The DP4+ calculations^15–17^ were carried out using the Excel spreadsheet available for free at sarotti-NMR.weebly.com.

#### ECD calculation

Conformation searches based on molecular mechanics with the MMFF94s force field were performed for (4*R*,5*S*,2′*S*)-1 (1a), (4*R*,5*R*,2′*R*)-1 (1c), (4*S*,5*S*,2′*S*)-1 (1e) and (4*S*,5*R*,2′*R*)-1 (1f), which gave 15, 16, 16 and 15 conformers with populations higher than 1%. All these conformers were further optimized by the density functional theory method at the B3LYP/6-31G(d) level in Gaussian 09 program package, which led to eleven (1a1 to 1a11), eleven (1c1 to 1c11), eleven (1e1 to 1e11) and eleven (1f1 to 1f11) conformers within a 2.0 kcal mol^−1^ energy threshold from global minimum, respectively. These predominant conformers were subjected to the theoretical calculation of ECD using time-dependent density functional theory (TDDFT) at the CAM-B3LYP/6-311+G (d,p) level in air. The calculated ECD curves for (4*R*,5*S*,2′*S*)-1 (1a), (4*R*,5*R*,2′*R*)-1 (1c), (4*S*,5*S*,2′*S*)-1 (1e), (4*S*,5*R*,2′*R*)-1 (1f) and their weighted ECD were all generated using SpecDis 1.60 with *σ* = 0.3 eV, and UV shift 2, respectively.^23^

## Conflicts of interest

There are no conflicts to declare.

## Supplementary Material

RA-008-C8RA04226H-s001
