# Supplementary material for: Albatredines A and B, a pair of epimers with unusual natural heterocyclic skeletons from edible mushroom Albatrellus confluens
Source: RSC Adv. 2018 Jul 2;8(42):23914–8. doi: 10.1039/c8ra04226h (PMC9081739; doi:10.1039/c8ra04226h)
Supplement: RA-008-C8RA04226H-s001 [file RA-008-C8RA04226H-s001.pdf]

Electronic Supplementary Material (ESI) for RSC Advances.
This journal is © The Royal Society of Chemistry 2018

Albatredine A and B, a pair of epimers with Unusual Natrural Hererocylic Skeletons from Edible Mushroom *Albatrellus confluens*

Shuaibing Zhang, Ying Huang, Shijun He, Zhenghui Li, Heping Chen, Bin Wu, Jianping Zuo, Tao Feng and Jikai Liu

Contents

1. Supplementary Figures

Figure S1. ^{1}H NMR spectrum in methanol-*d*$_4$ for Compound **1**

Figure S2. ^{13}C NMR and DEPT spectrum in methanol-*d*$_4$ for Compound **1**

Figure S3. ^{1}H-^{1}H COSY spectrum in methanol-*d*$_4$ for Compound **1**

Figure S4. HSQC spectrum in methanol-*d*$_4$ for Compound **1**

Figure S5. HMBC spectrum in methanol-*d*$_4$ for Compound **1**

Figure S6. ^{1}H NMR spectrum in DMSO-*d*$_6$ for Compound **1**

Figure S7. ^{13}C NMR spectrum in DMSO-*d*$_6$ for Compound **1**

Figure S8. ^{1}H-^{1}H COSY spectrum in DMSO-*d*$_6$ for Compound **1**

Figure S9. HMBC spectrum in DMSO-*d*$_6$ for Compound **1**

Figure S10. HRESIMS spectrum for Compound **1**

Figure S11. ^{1}H NMR spectrum in methanol-*d*$_4$ for Compound **2**

Figure S12. ^{13}C NMR and DEPT spectrum in methanol-*d*$_4$ for Compound **2**

Figure S13. ^{1}H-^{1}H COSY spectrum in methanol-*d*$_4$ for Compound **2**

Figure S14. HSQC spectrum in methanol-*d*$_4$ for Compound **2**

Figure S15. HMBC spectrum in methanol-*d*$_4$ for Compound **2**

Figure S16. HRESIMS spectrum for Compound **2**

2. Computational details for **1** and **2**

2.1 ^{13}C NMR calculation details for **1** and **2**

Figure S17. Optimized conformers of **1a** (**1a1-1a11**)

Table S1. Energy analysis for conformers of **1a1-1a11** at B3LYP/6-31G(d) level in the gas phase

Figure S18. Optimized conformers of **1b** (**1b1-1b12**).

Table S2. Energy analysis for conformers of **1b1-1b112** at B3LYP/6-31G(d) level in the gas phase

Figure S19. Optimized conformers of **1c** (**1c1-1c11**).

Table S3. Energy analysis for conformers of **1c1-1c11** at B3LYP/6-31G(d) level in the gas phase

Figure S20. Optimized conformers of **1d** (**1d1-1d10**).

Table S4. Energy analysis for conformers of **1d1-1d10** at B3LYP/6-31G(d) level in the gas phase

Table S5. ^{13}C NMR calculation details for **1a**

Table S6. ^{1}H NMR calculation details for **1a**

Table S7. ^{13}C NMR calculation details for **1b**

Table S8. ^{1}H NMR calculation details for **1b**

Table S9. ^{13}C NMR calculation details for **1c**

Table S10. ^{1}H NMR calculation details for **1c**

Table S11. ^{13}C NMR calculation details for **1d**

Table S12. ^{1}H NMR calculation details for **1d**

Figure S21. Left: Regression analysis of experimental versus calculated ^{13}C NMR chemical shifts of **1** at mPW1PW91/6-311g(d,p) level; linear fitting was shown as a line; Right: Relative chemical shift errors between scaled ^{13}C NMR and experimental ^{13}C NMR for **1** (δ_{corr} obtained by linear fit δ_{exp} versus δ_{calcd}).

Figure S22. Left: Regression analysis of experimental versus calculated ^{1}H NMR chemical shifts of **1** at mPW1PW91/6-311g(d,p) level; linear fitting was shown as a line; Right: Relative chemical shift errors between scaled ^{1}H NMR and experimental ^{1}H NMR for **1** (δ_{corr} obtained by linear fit δ_{exp} versus δ_{calcd}).

Figure S23. Left: Regression analysis of experimental versus calculated ^{13}C NMR chemical shifts of **2** at mPW1PW91/6-311g(d,p) level; linear fitting was shown as a line; Right: Relative chemical shift errors between scaled ^{13}C NMR and experimental ^{13}C NMR for **2** (δ_{corr} obtained by linear fit δ_{exp} versus δ_{calcd}).
Figure S24. Left: Regression analysis of experimental versus calculated ^{1}H NMR chemical shifts of **2** at mPW1PW91/6-311g(d,p) level; linear fitting was shown as a line; Right: Relative chemical shift errors between scaled ^{1}H NMR and experimental ^{1}H NMR for **2** (δ_{corr} obtained by linear fit δ_{exp} versus δ_{calcd}).
Table S13. The DP4+ probabilities of **1a**, **1b**, **1c** and **1d**
2.2 ECD calculation details for **1a**, **1c, 1e** and **1f**
Figure S25. Optimized conformers of **1e** (**1e1-1e11**).
Table S14. Energy analysis for conformers of **1e1-1e11** at B3LYP/6-31G(d) level in the gas phase
Figure S26. Optimized conformers of **1f (1f1-1f11**).
Table S15. Energy analysis for conformers of **1f1-1f11** at B3LYP/6-31G(d) level in the gas phase
Figure S27. Comparison of the calculated ECD spectra with the experimental spectrum.
2.3 Determination of the substitution pattern.
Figure S28. Optimized conformers of **1g** (**1g1-1g10**).
Table S16. Energy analysis for conformers of **1g1-1g10** at B3LYP/6-31G(d) level in the gas phase
Figure S29. Optimized conformers of **1h** (**1h1-1h3**).
Table S17. Energy analysis for conformers of **1h1-1h3** at B3LYP/6-31G(d) level in the gas phase
Table S18. ^{13}C NMR calculation details for **1g**
Table S19. ^{13}C NMR calculation details for **1h**
Table S20. The DP4+ probabilities of **1a**, **1g** and **1h**.
3. References

1. Supplementary Figures

Figure S1. ^{1}H NMR spectrum in methanol-d_4 for Compound **1**

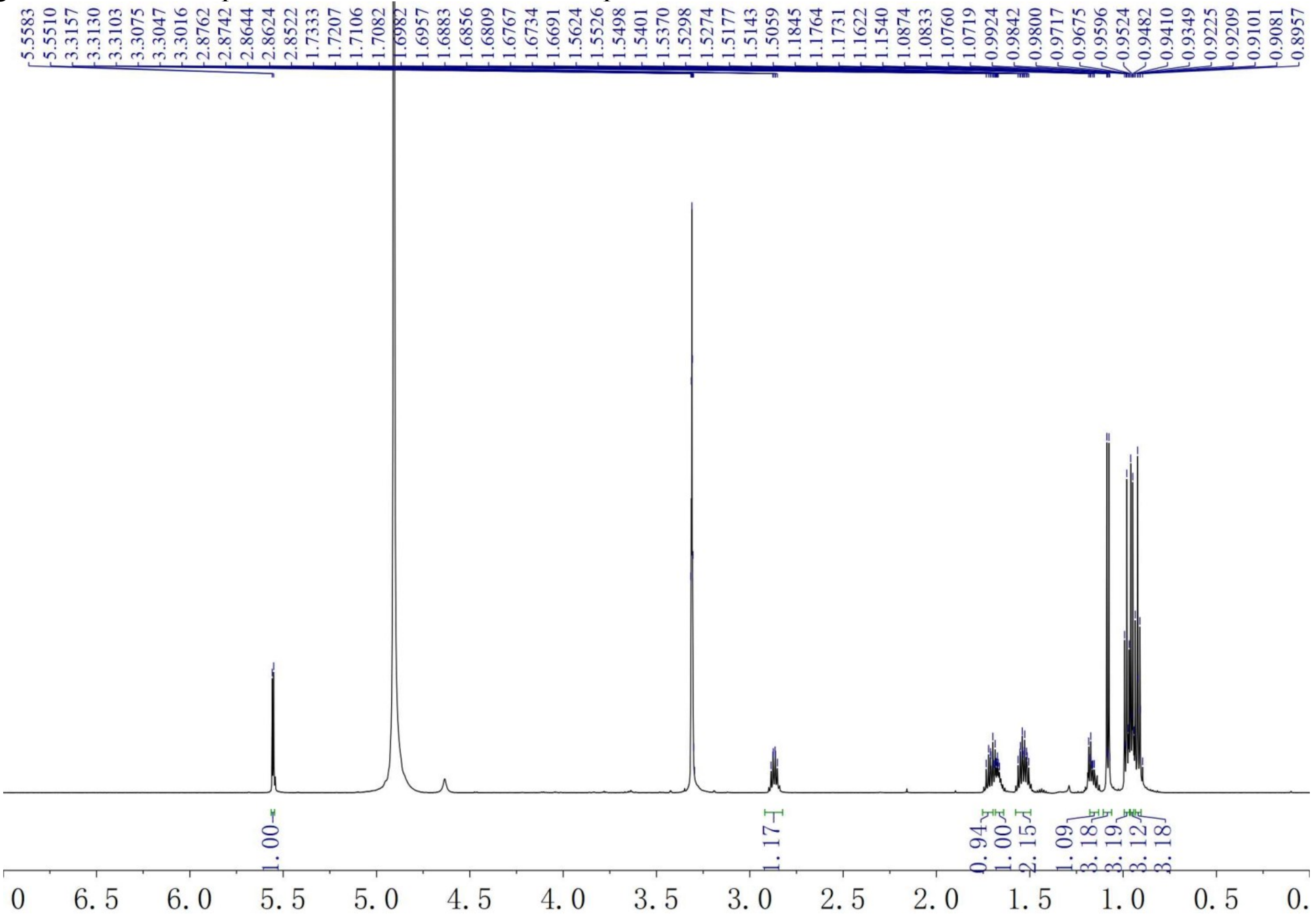

Figure S2. ^{13}C NMR and DEPT spectrum in methanol-d_4 for Compound **1**

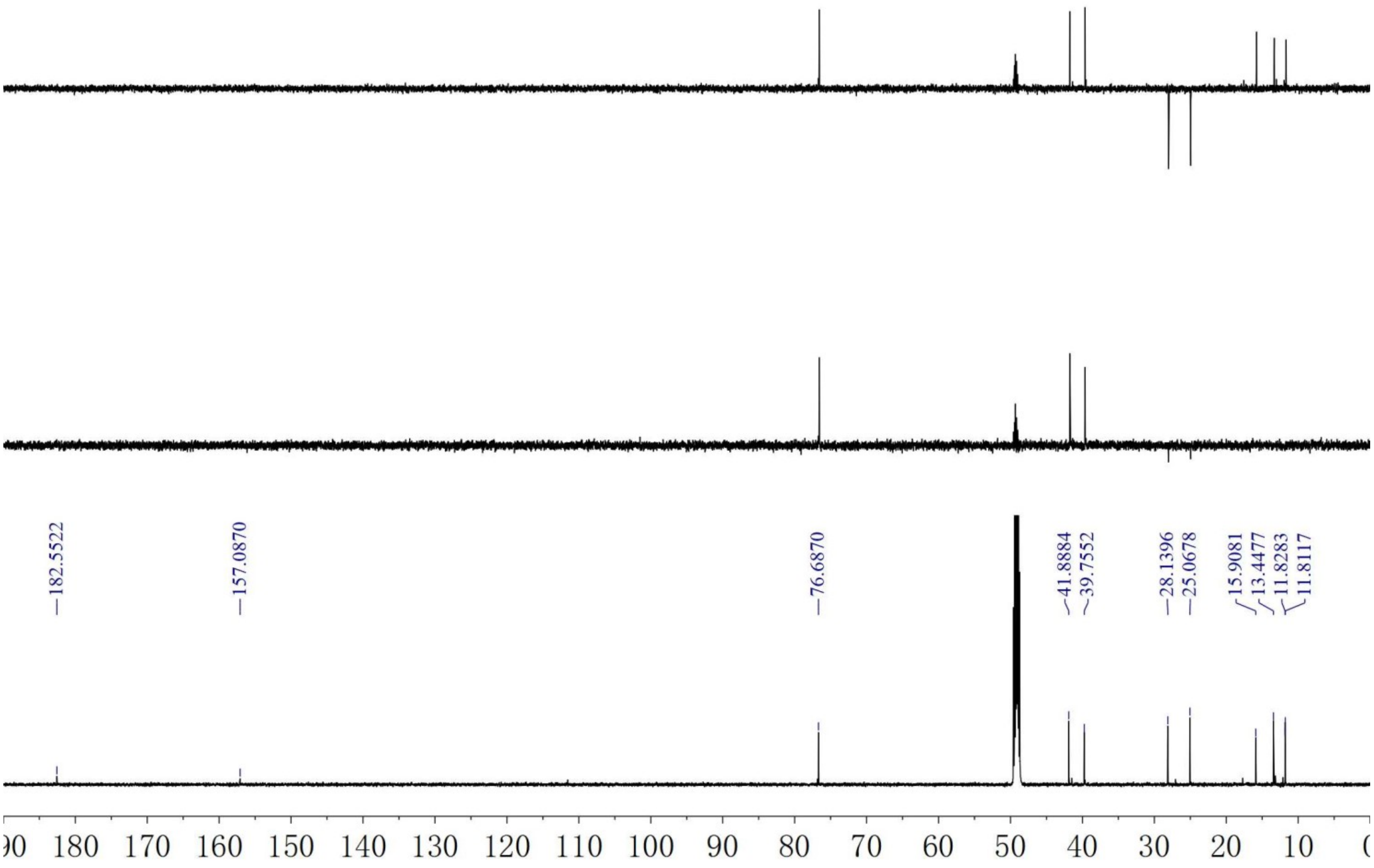

Figure S3. ^{1}H-^{1}H COSY spectrum in methanol-d_4 for Compound **1**

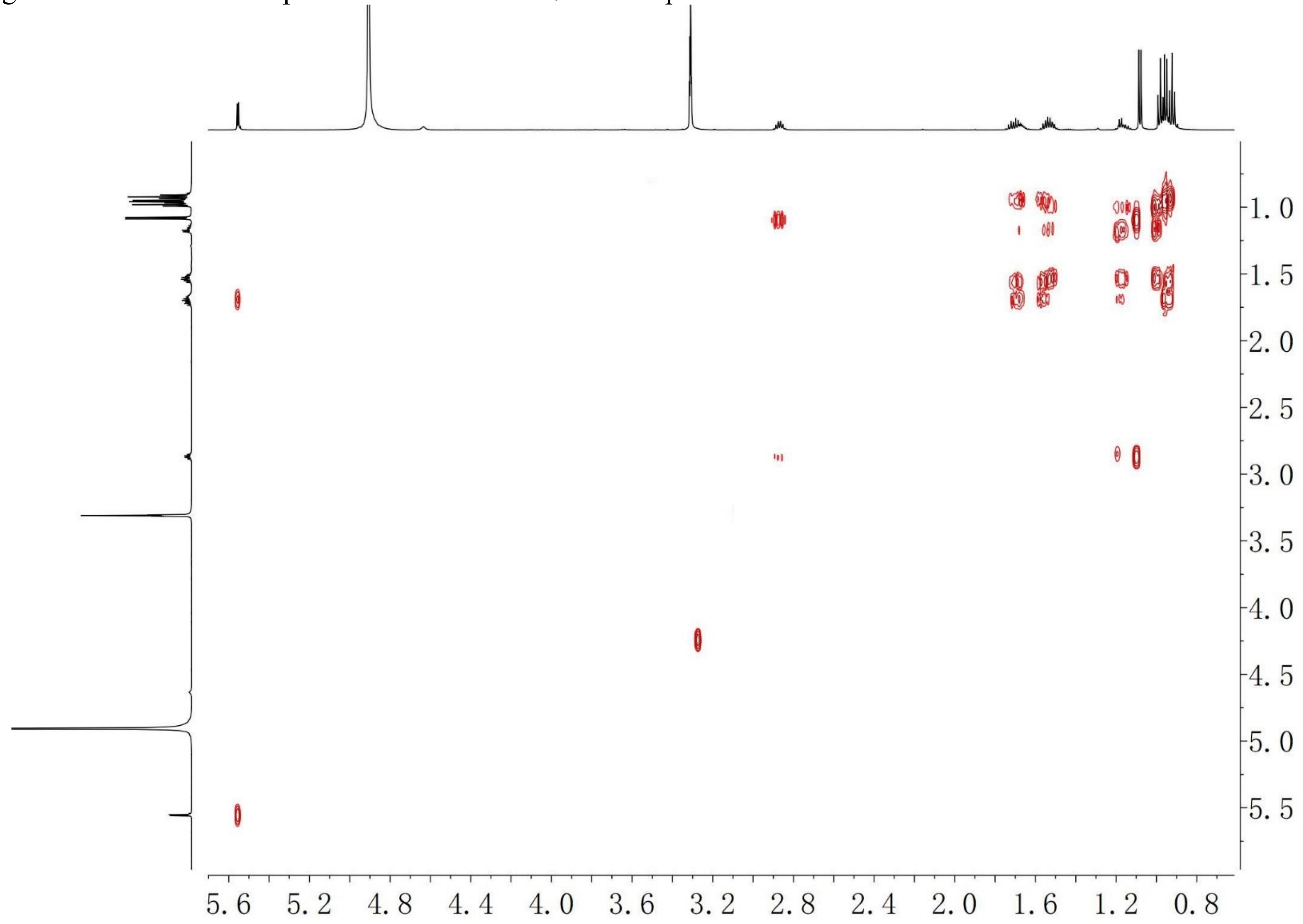

Figure S4. HSQC spectrum in methanol-d_4 for Compound **1**

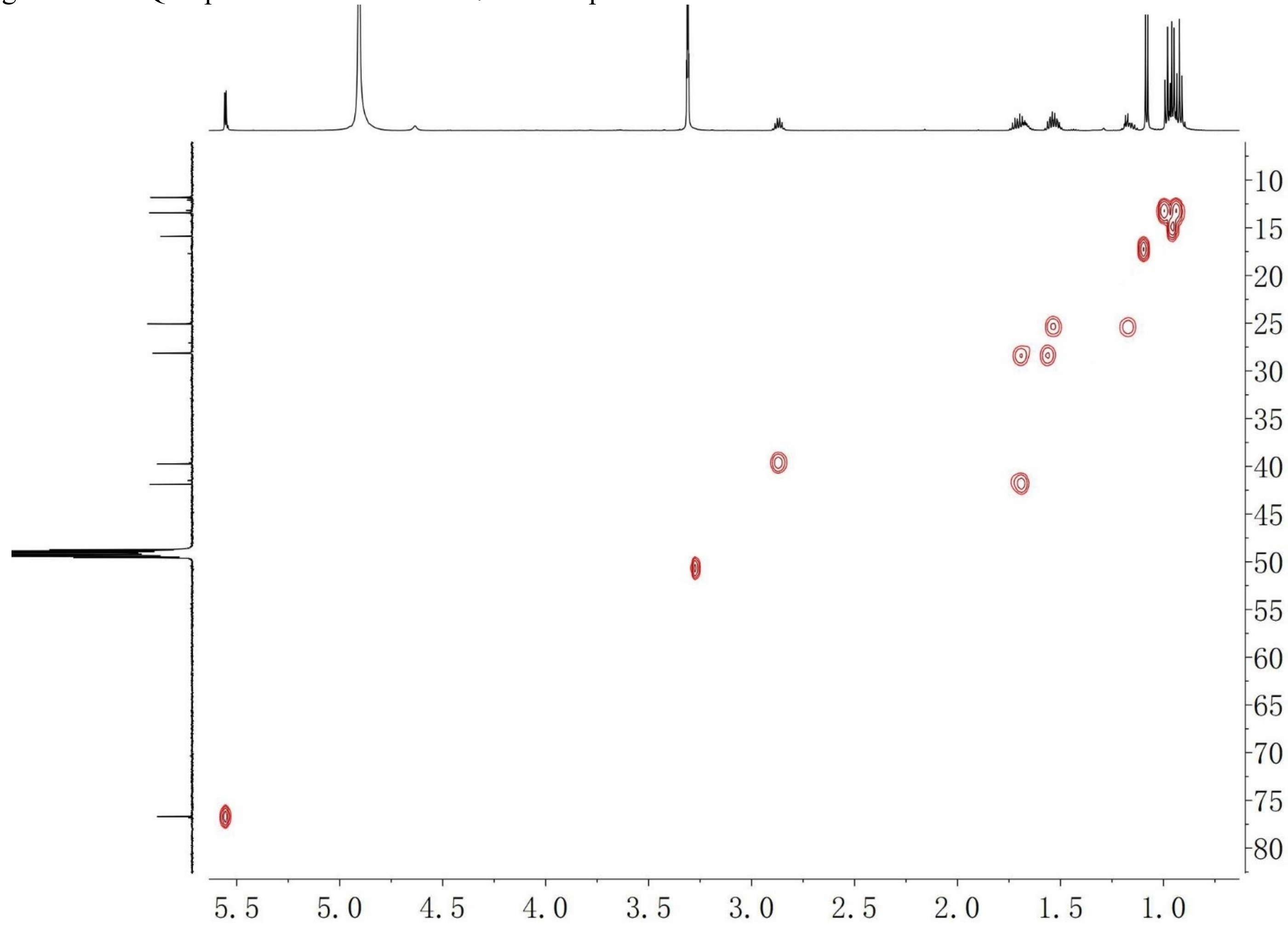

Figure S5. HMBC spectrum in methanol-d_4 for Compound **1**

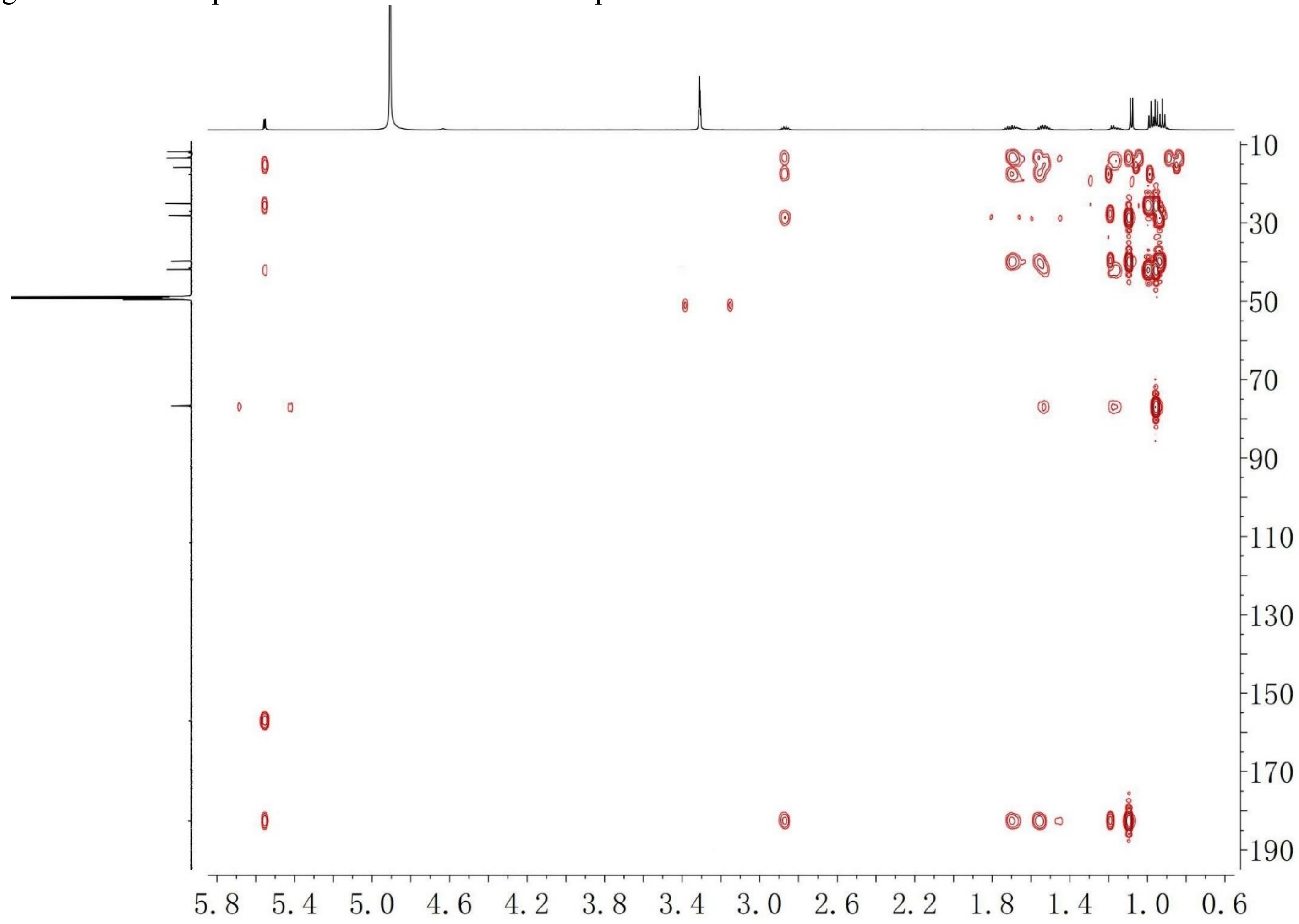

Figure S6. ^{1}H NMR spectrum in DMSO-d_6 for Compound **1**

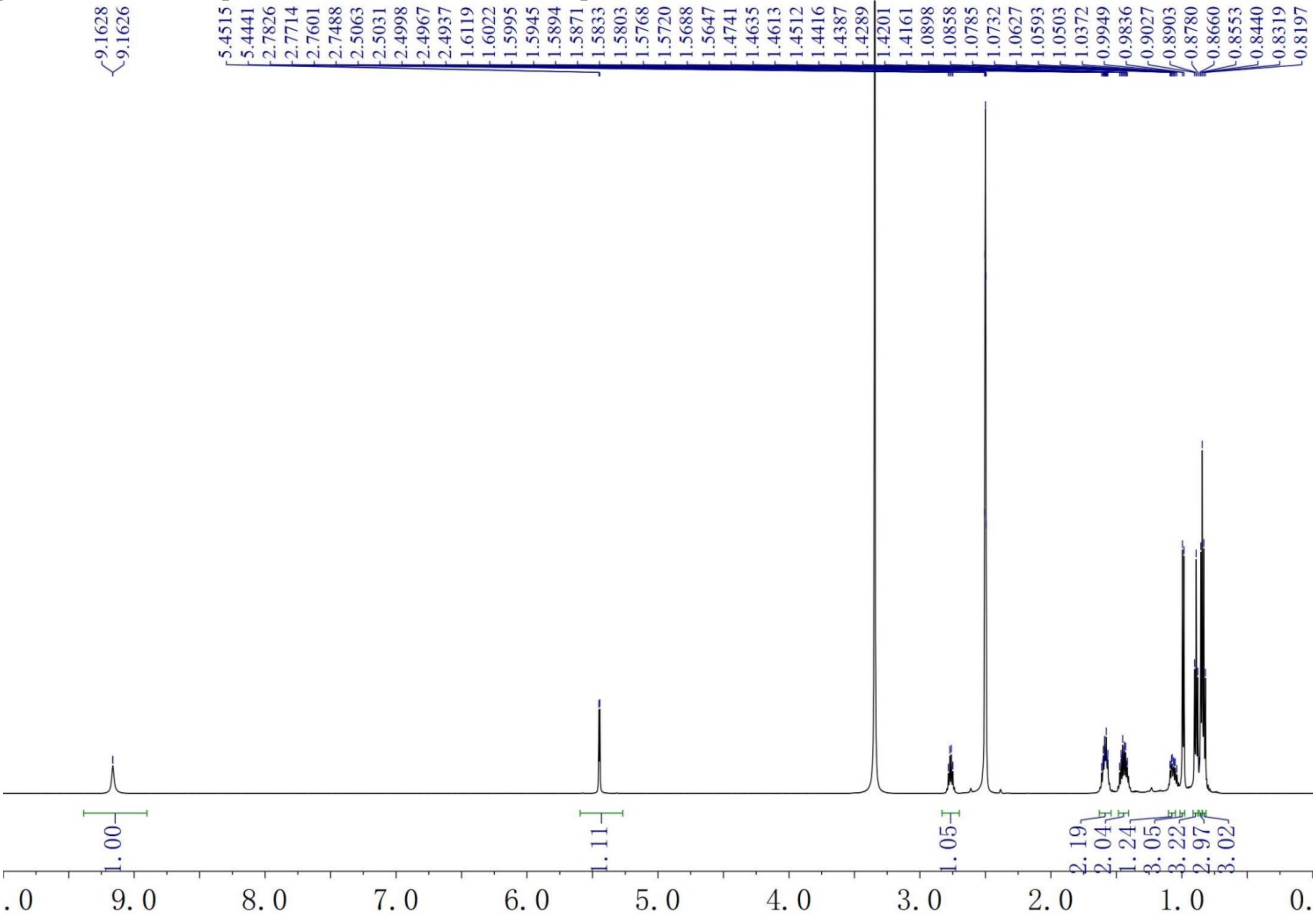

Figure S7. ^{13}C NMR spectrum in DMSO-d_6 for Compound **1**

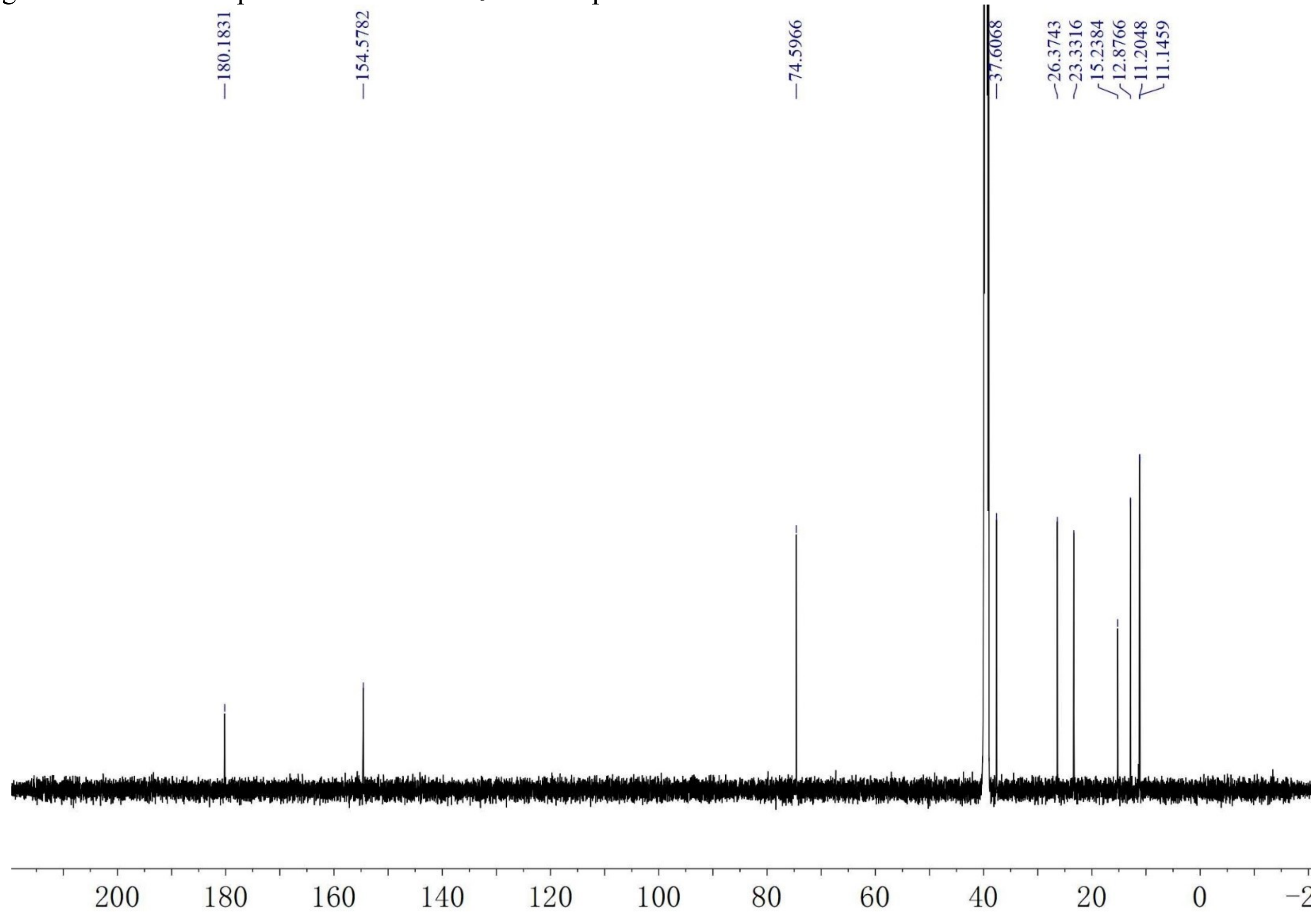

Figure S8. ^{1}H-^{1}H COSY spectrum in DMSO-d_6 for Compound **1**

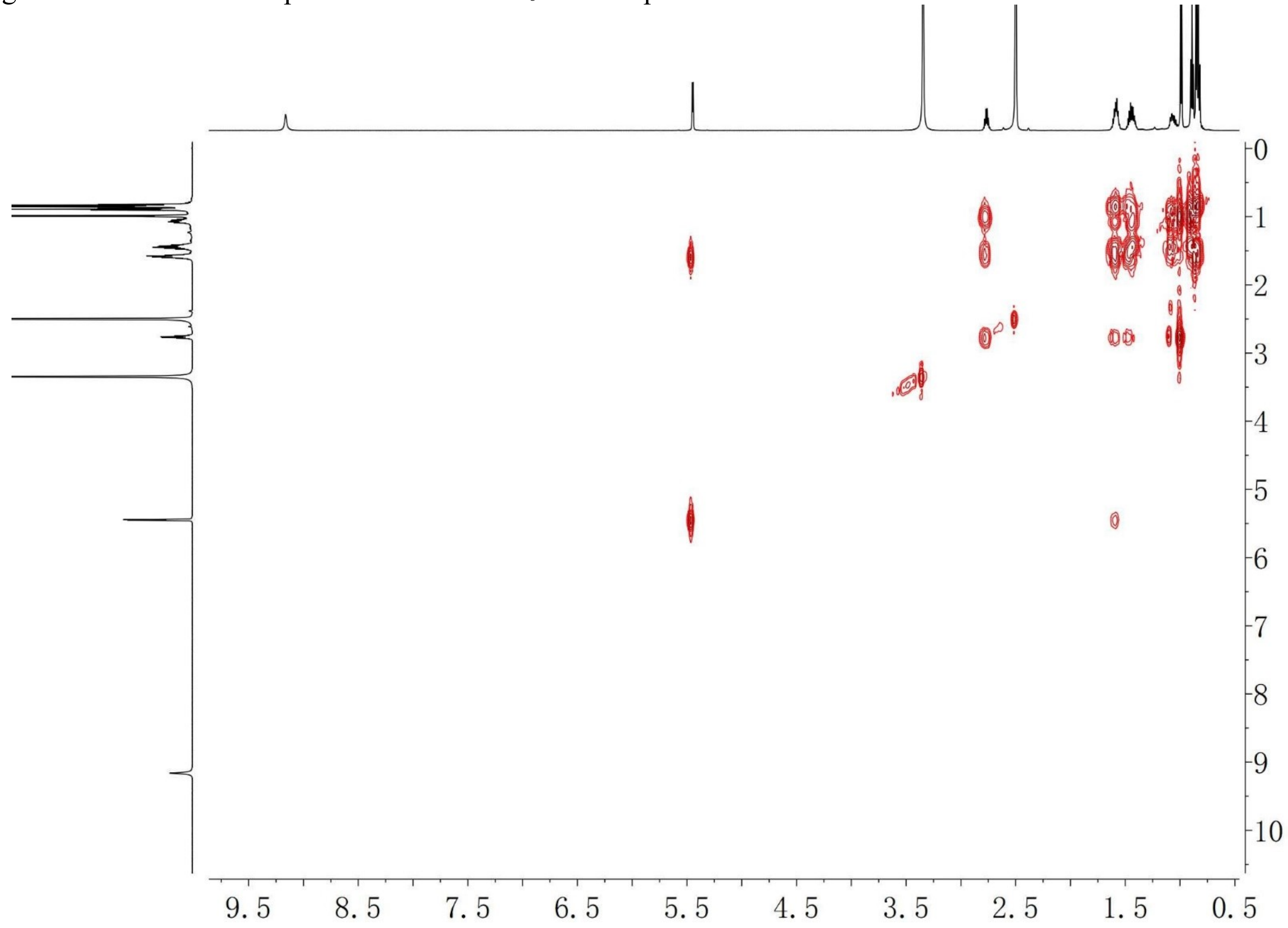

Figure S9. HMBC spectrum in DMSO-d_6 for Compound **1**

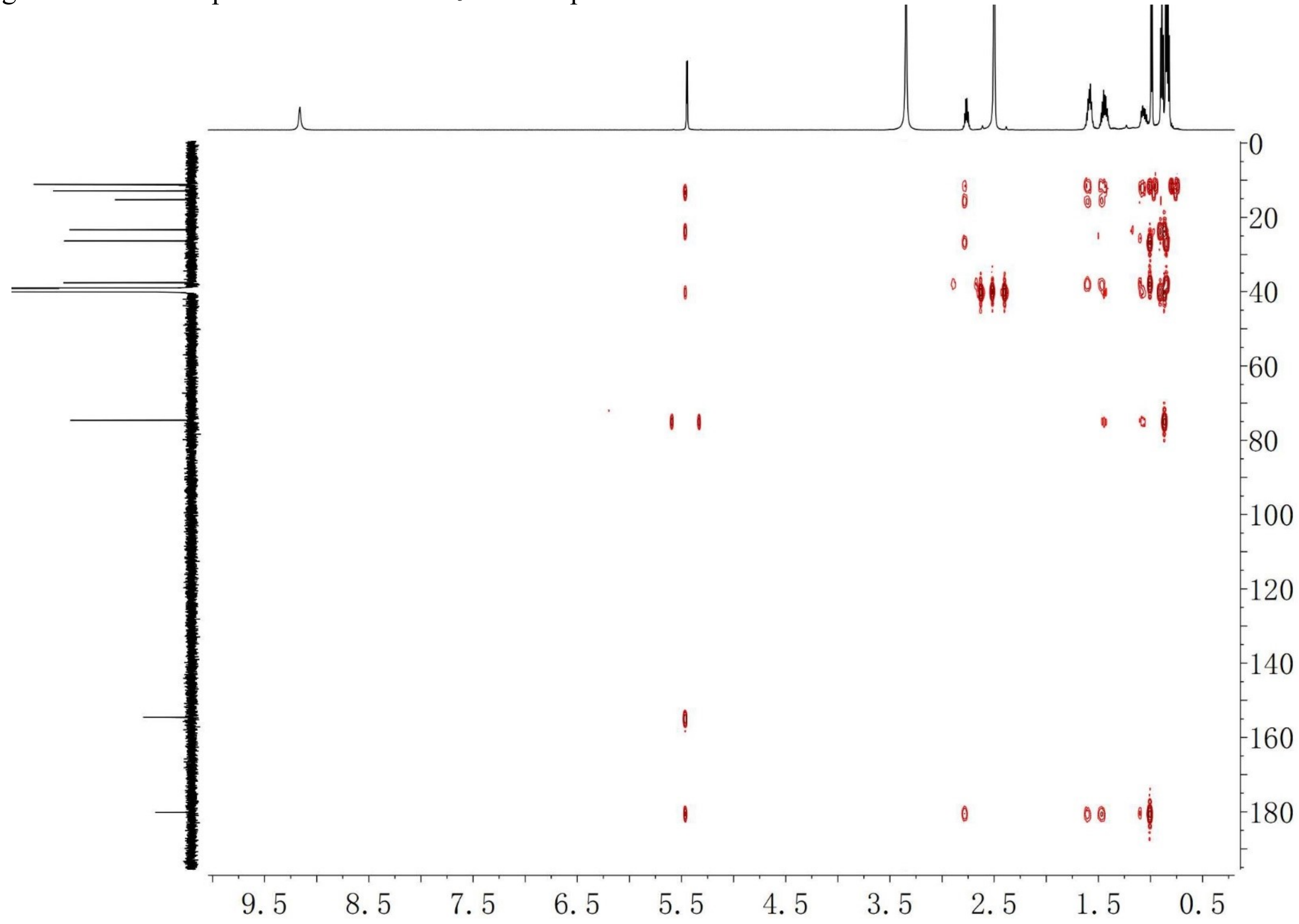

Figure S10. HRESIMS spectrum for Compound **1**

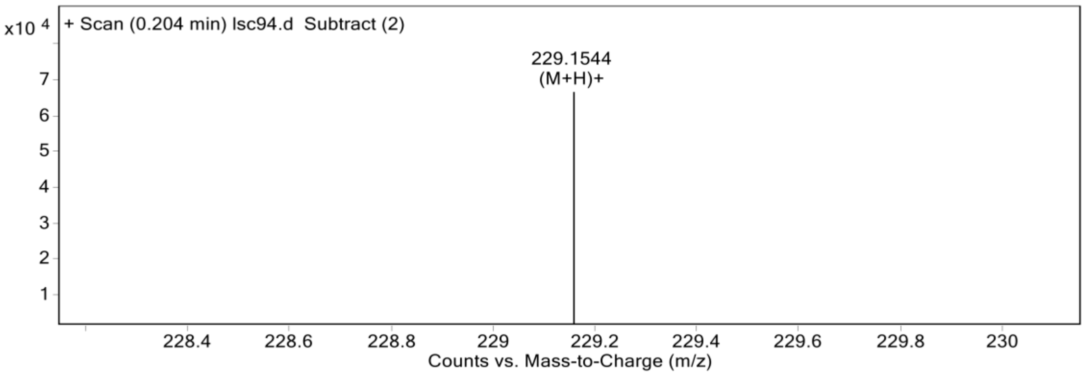

Peak List

m/z	z	Abund	Formula	Ion
118.0864	1	7392.96		
130.0866	1	23912.34		
229.1544	1	67032.44	C11 H20 N2 O3	(M+H)+
230.1581	1	9165.5	C11 H20 N2 O3	(M+H)+
251.1365		25167.27		
267.1106	1	19084.79		
303.1911	1	9017.3		
313.1069	1	12935.8		

Formula Calculator Element Limits

Element	Min	Max
C	3	60
H	0	120
O	0	30
N	0	10

Formula Calculator Results

Formula	CalculatedMass	CalculatedMz	Mz	Diff. (mDa)	Diff. (ppm)	DBE
C11 H20 N2 O3	228.1474	229.1547	229.1544	0.1	0.5	3.0000

Figure S11. ^{1}H NMR spectrum in methanol-d_4 for Compound **2**

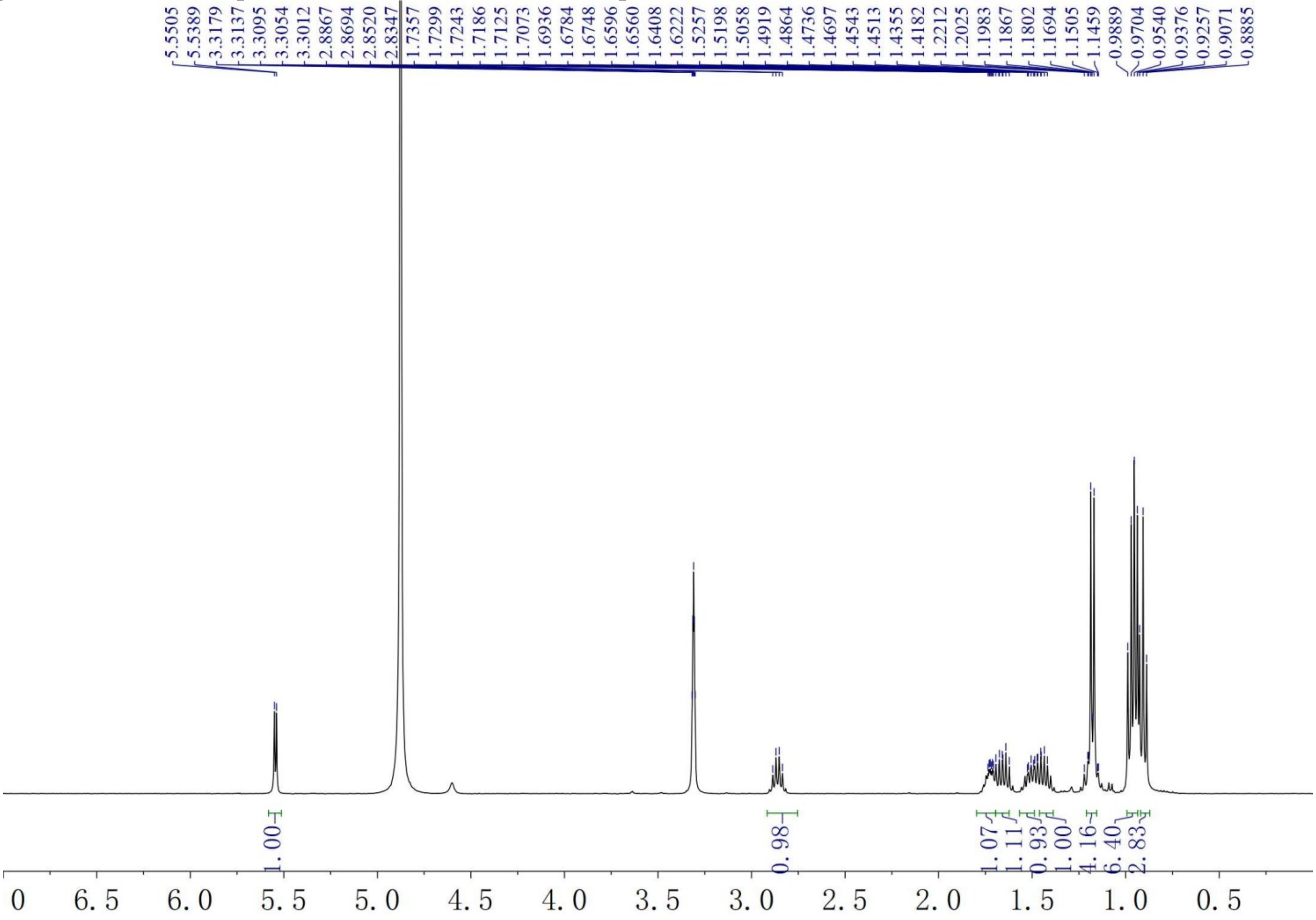

Figure S12. ^{13}C NMR and DEPT spectrum in methanol-d_4 for Compound **2**

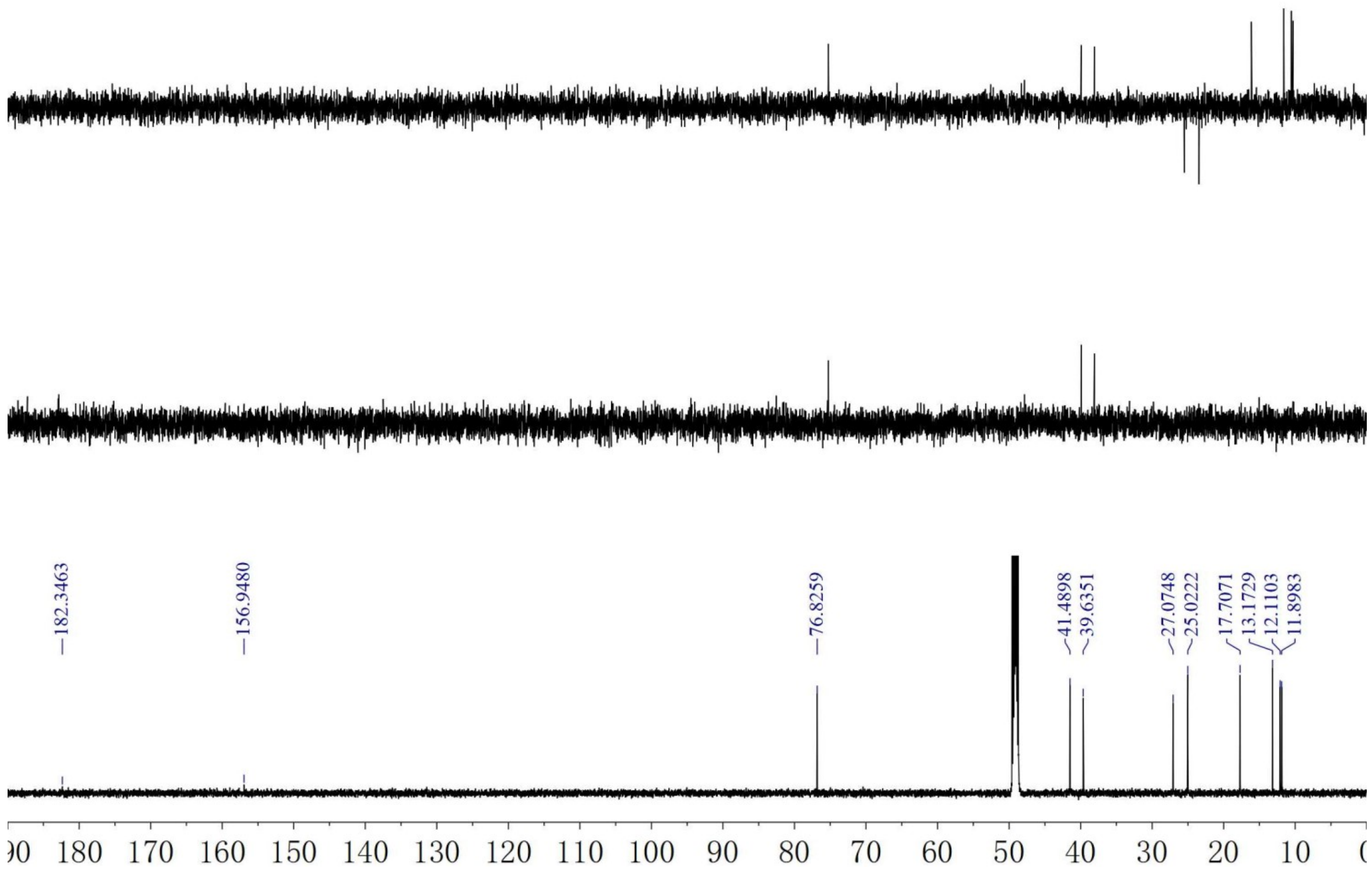

Figure S13. ^1H-^1H COSY spectrum in methanol-d_4 for Compound **2**

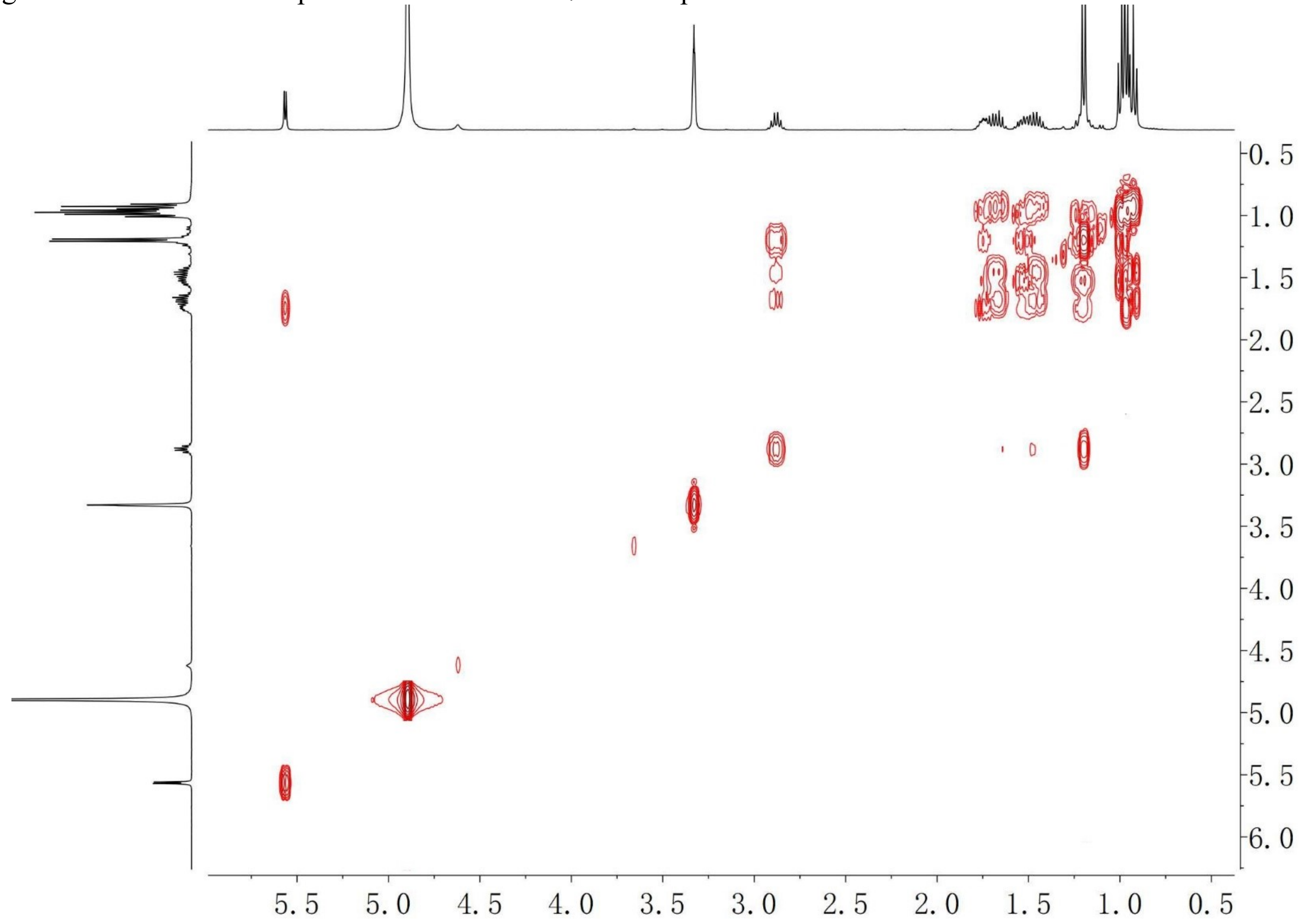

Figure S14. HSQC spectrum in methanol-d_4 for Compound **2**

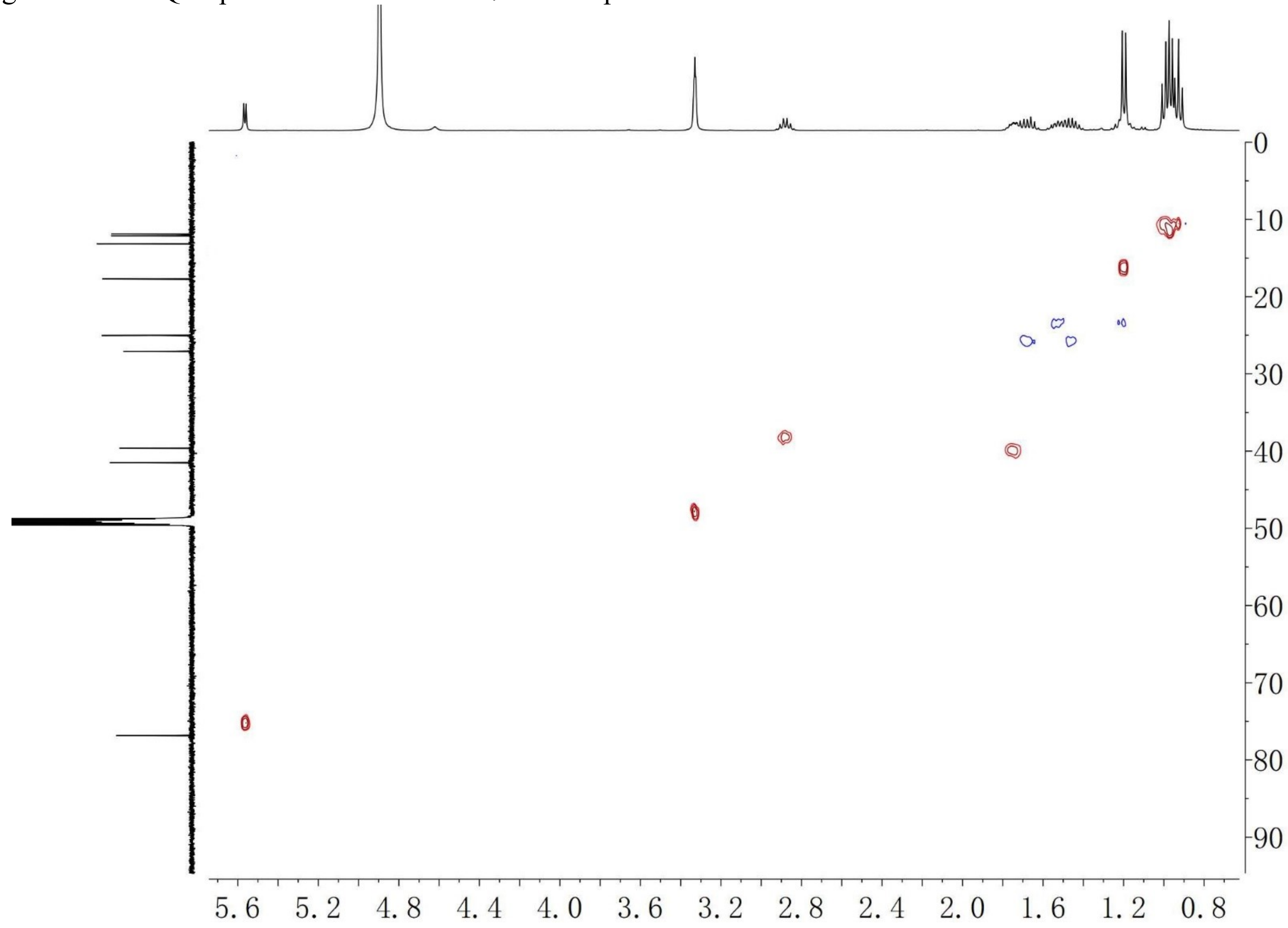

Figure S15. HMBC spectrum in methanol-d_4 for Compound **2**

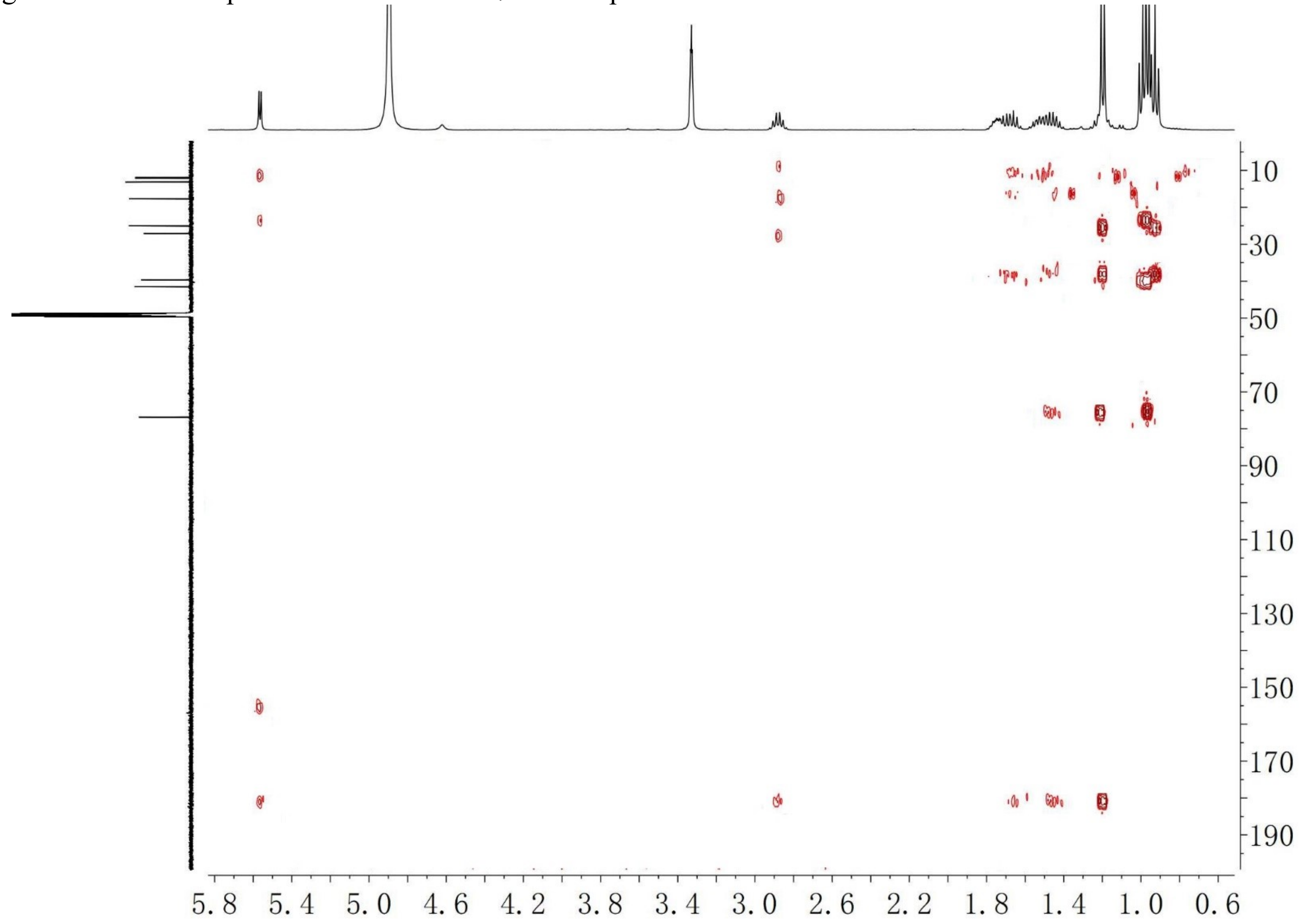

Figure S16. HRESIMS spectrum for Compound **2**

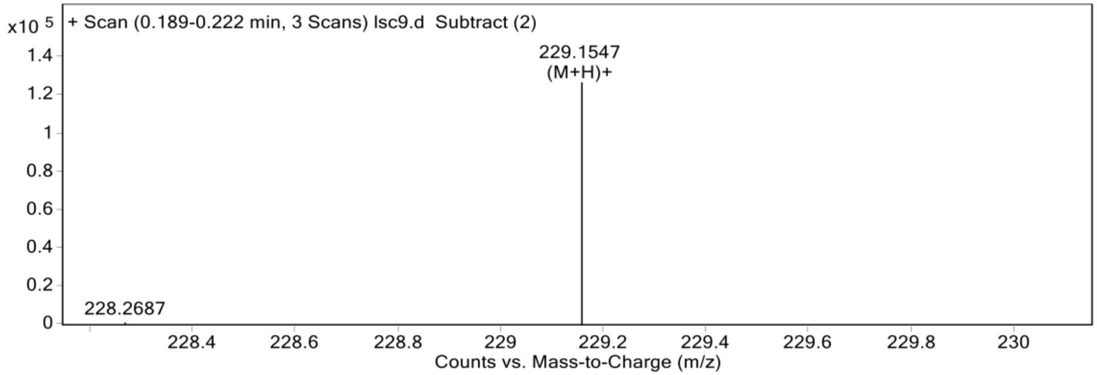

Peak List

m/z	z	Abund	Formula	Ion
86.0968		9631.73		
118.0863		15697.1		
130.0866	1	51371.19		
229.1547	1	127253.02	C11 H20 N2 O3	(M+H)+
230.1579	1	17038.29	C11 H20 N2 O3	(M+H)+
251.1366	1	40505.64		
267.1106	1	33710.73		
313.1073	1	17853.14		

Formula Calculator Element Limits

Element	Min	Max
C	3	60
H	0	120
O	0	30
N	0	10

Formula Calculator Results

Formula	CalculatedMass	CalculatedMz	Mz	Diff. (mDa)	Diff. (ppm)	DBE
C11 H20 N2 O3	228.1474	229.1547	229.1547	-0.1	-0.3	3.0000

2. Computational details for 1 and 2

2.1 ^{13}C NMR calculation details for **1** and **2**

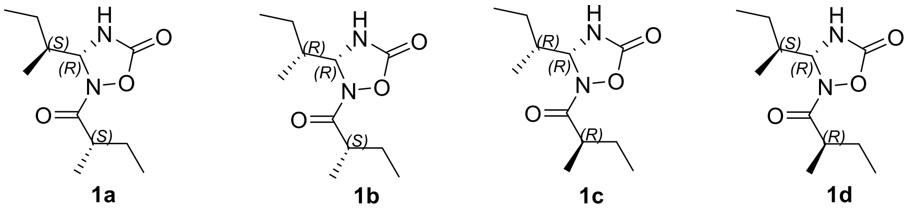

Conformation searches based on molecular mechanics with MMFF94s force field were performed for (4*R*,5*S*,2′*S*)-**1** (**1a**), (4*R*,5*R*,2′*S*)-**1** (**1b**), (4*R*,5*R*,2′*R*)-**1** (**1c**) and (4*R*,5*S*,2′*R*)-**1** (**1d**) have 15, 15, 16 and 15 conformers with populations higher than 1%. respectively[1-2]. All these conformers were further optimized by the density functional theory method at the B3LYP/6-31G(d) level in Gaussian 09 program package[3], led to eleven (**1a1** to **1a11**), twelve (**1b1** to **1b12**), eleven (**1c1** to **1c11**) and ten (**1d1** to **1d10**) conformers within a 2.0 kcal/mol energy threshold from global minimum, respectively. These predominant conformers were subjected to NMR calculation. Gauge-Independent Atomic Orbital (GIAO) calculations of ^{13}C NMR of the conformers were accomplished by density functional theory (DFT) at mPW1PW91/6-311g(d,p) level in methanol with PCM model. The ^{13}C NMR chemical shift of TMS was calculated in the same level and used as reference. The calculated NMR data of these conformers were averaged according to the Boltzmann distribution theory and their relative Gibbs free energy.[4]

Figure S17. Optimized conformers of **1a** (**1a1-1a11**)

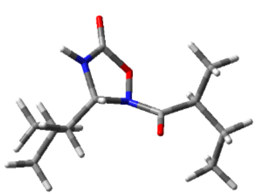

1a1

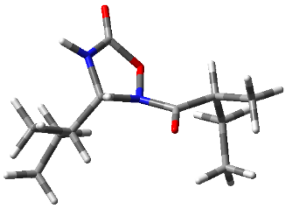

1a2

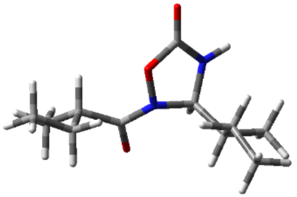

1a3

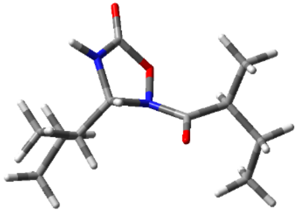

1a4

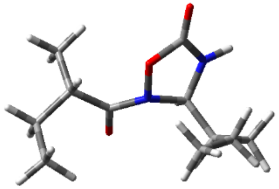

1a5

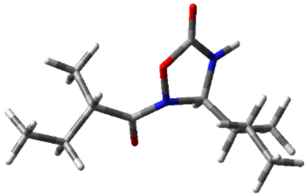

1a6

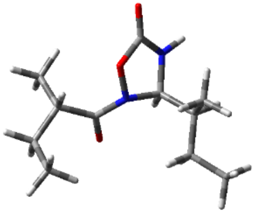

1a7

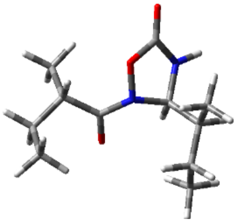

1a8

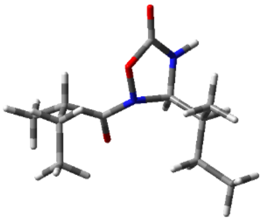

1a9

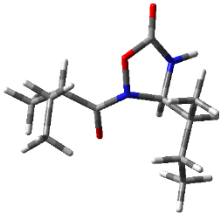

1a10

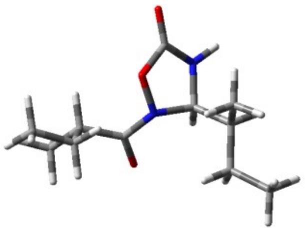

1a11

Table S1. Energy analysis for conformers of **1a1-1a11** at B3LYP/6-31G(d) level in the gas phase

species	$E'=E+ZPE$	E	H	G	ΔE (kcal/mol)	P_E%
**1a1**	-766.295543	-766.277272	-766.276328	-766.342701	0.3495175	14.99%
**1a2**	-766.294309	-766.276084	-766.27514	-766.34158	1.052945	4.57%
**1a3**	-766.294787	-766.276405	-766.275461	-766.342587	0.4210525	13.28%
**1a4**	-766.295546	-766.277274	-766.27633	-766.342706	0.34638	15.07%
**1a5**	-766.293612	-766.275183	-766.274239	-766.341678	0.99145	5.07%
**1a6**	-766.294722	-766.276177	-766.275233	-766.343258	0	27.04%
**1a7**	-766.295408	-766.27729	-766.276346	-766.342088	0.734175	7.83%
**1a8**	-766.295143	-766.277123	-766.276179	-766.341307	1.2242525	3.42%
**1a9**	-766.294225	-766.276172	-766.275228	-766.340852	1.509765	2.11%
**1a10**	-766.293959	-766.275991	-766.275047	-766.34034	1.831045	1.23%
**1a11**	-766.294615	-766.276479	-766.275535	-766.341734	0.95631	5.38%

E, *E'*, *H*, *G*: total energy, total energy with zero point energy (ZPE), enthalpy, and Gibbs free energy

Figure S18. Optimized conformers of **1b** (**1b1-1b12**).

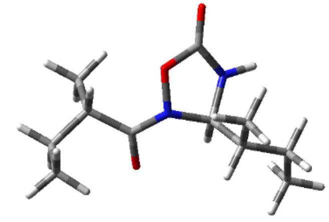

1b1

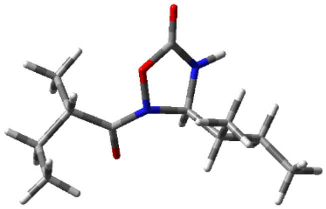

1b2

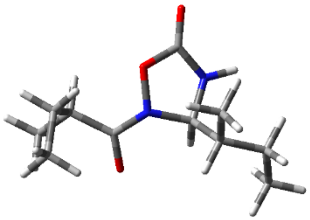

1b3

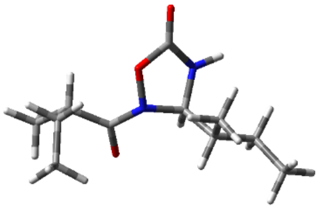

1b4

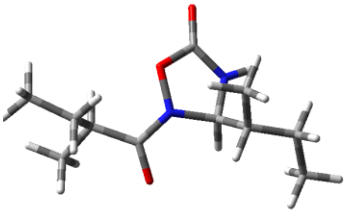

1b5

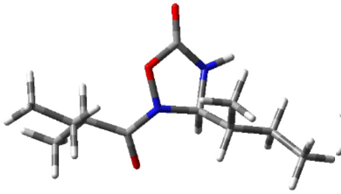

1b6

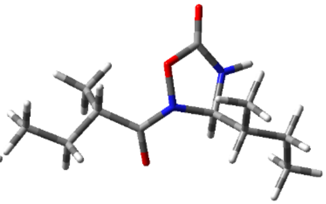

1b7

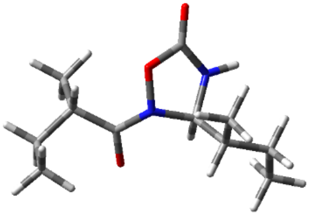

1b8

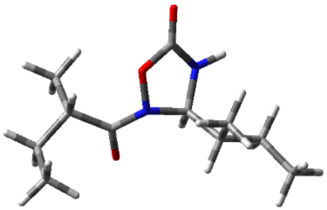

1b9

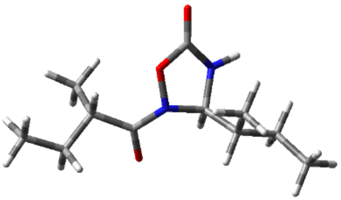

1b10

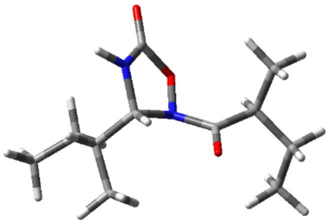

1b11

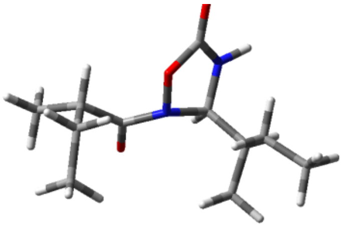

1b12

Table S2. Energy analysis for conformers of **1b1-1b112** at B3LYP/6-31G(d) level in the gas phase

species	*E'*=*E*+*ZPE*	*E*	*H*	*G*	*ΔE* (kcal/mol)	P_E%
**1b1**	-766.29451	-766.276331	-766.275386	-766.341469	0.44176	10.07%
**1b2**	-766.295117	-766.276896	-766.275952	-766.342173	0	21.23%
**1b3**	-766.293086	-766.274925	-766.273981	-766.340308	1.1702875	2.94%
**1b4**	-766.293651	-766.275488	-766.274544	-766.340628	0.9694875	4.13%
**1b5**	-766.29363	-766.275402	-766.274458	-766.340953	0.76555	5.83%
**1b6**	-766.294163	-766.275939	-766.274995	-766.341401	0.48443	9.37%
**1b7**	-766.293066	-766.274893	-766.273949	-766.340062	1.3246525	2.27%
**1b8**	-766.29451	-766.276331	-766.275387	-766.341468	0.4423875	10.06%
**1b9**	-766.295116	-766.276896	-766.275952	-766.342173	0	21.23%
**1b10**	-766.293761	-766.275496	-766.274552	-766.340962	0.7599025	5.89%
**1b11**	-766.294364	-766.276308	-766.275364	-766.340889	0.80571	5.45%
**1b12**	-766.292952	-766.274889	-766.273945	-766.339684	1.5618475	1.52%

E, *E'*, *H*, *G*: total energy, total energy with zero point energy (ZPE), enthalpy, and Gibbs free energy

Figure S19. Optimized conformers of **1c** (**1c1-1c11**).

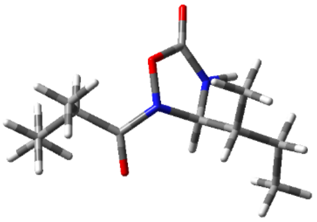

1c1

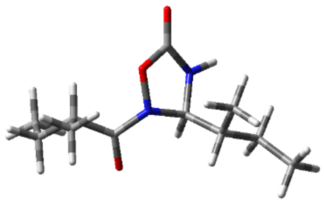

1c2

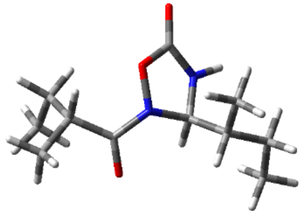

1c3

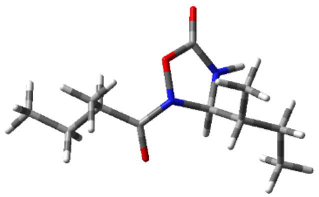

1c4

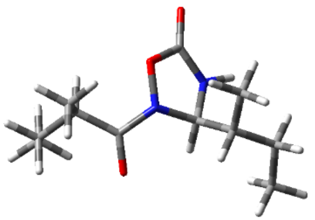

1c5

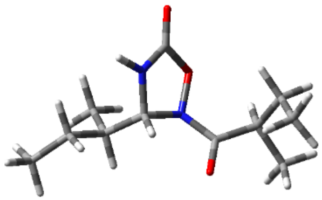

1c6

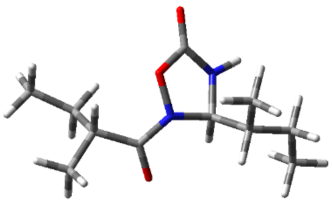

1c7

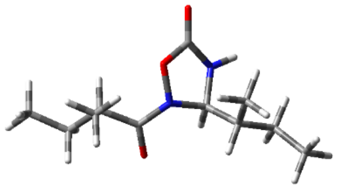

1c8

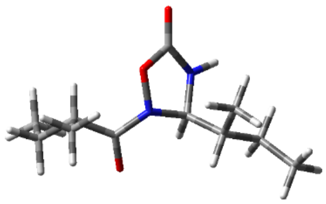

1c9

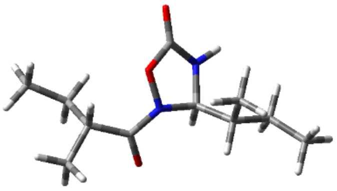

1c10

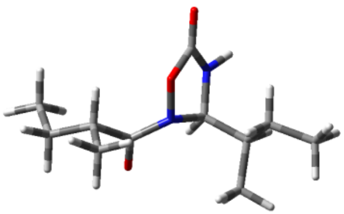

1c11

Table S3. Energy analysis for conformers of **1c1-1c11** at B3LYP/6-31G(d) level in the gas phase

species	E'=E+ZPE	E	H	G	ΔE (kcal/mol)	P_E%
**1c1**	-766.294863	-766.276687	-766.275743	-766.294863	0.6093025	6.66%
**1c2**	-766.295448	-766.277213	-766.276269	-766.295448	0.013805	18.21%
**1c3**	-766.293619	-766.275347	-766.274403	-766.293619	1.218605	2.38%
**1c4**	-766.293947	-766.275734	-766.27479	-766.293947	1.1916225	2.49%
**1c5**	-766.294806	-766.276534	-766.27559	-766.294806	0.3545375	10.24%
**1c6**	-766.294132	-766.275843	-766.274899	-766.294132	0.8603025	4.36%
**1c7**	-766.294391	-766.276007	-766.275062	-766.294391	0.530865	7.61%
**1c8**	-766.294706	-766.276311	-766.275367	-766.294706	0.2942975	11.34%
**1c9**	-766.295326	-766.27702	-766.276076	-766.295326	0	18.64%
**1c10**	-766.294868	-766.276498	-766.275554	-766.294868	0.2428425	12.37%
**1c11**	-766.29498	-766.276855	-766.275911	-766.29498	0.7021725	5.70%

E, *E'*, *H*, *G*: total energy, total energy with zero point energy (ZPE), enthalpy, and Gibbs free energy

Figure S20. Optimized conformers of **1d** (**1d1-1d10**).

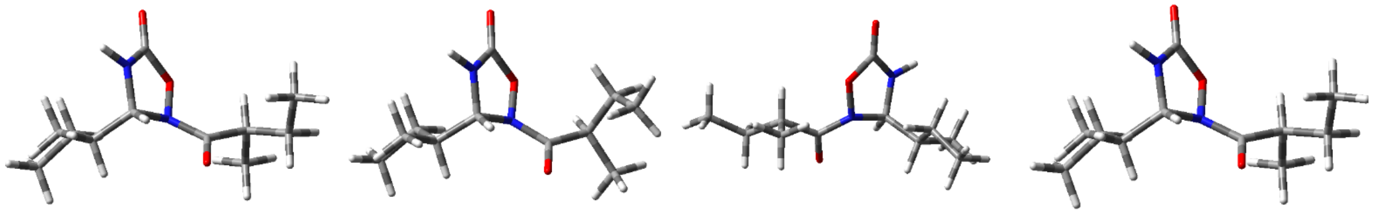

1d1 **1d2** **1d3** **1d4**

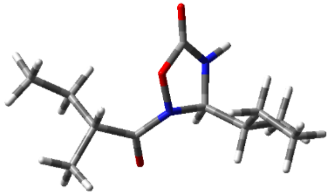

1d5

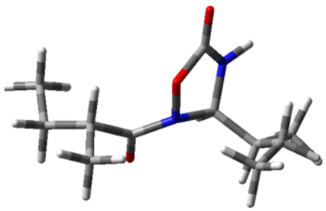

1d6

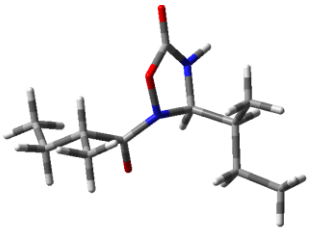

1d7

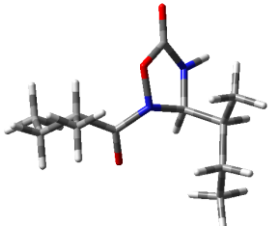

1d8

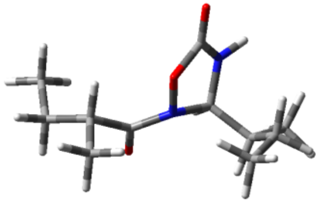

1d9

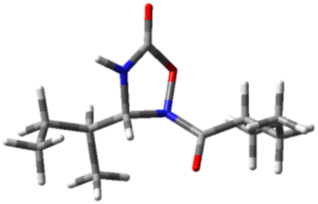

1d10

Table S4. Energy analysis for conformers of **1d1-1d10** at B3LYP/6-31G(d) level in the gas phase

species	E'=E+ZPE	E	H	G	ΔE (kcal/mol)	P_E%
**1d1**	-766.294565	-766.276324	-766.27538	-766.342002	0.003765	22.52%
**1d2**	-766.292685	-766.274466	-766.273522	-766.339987	1.2681775	2.66%
**1d3**	-766.293602	-766.275302	-766.274358	-766.341223	0.4925875	9.87%
**1d4**	-766.294565	-766.276324	-766.27538	-766.342008	0	22.66%
**1d5**	-766.293854	-766.275606	-766.274662	-766.341074	0.586085	8.43%
**1d6**	-766.292268	-766.274115	-766.273171	-766.339484	1.58381	1.56%
**1d7**	-766.294363	-766.276285	-766.27534	-766.341331	0.4248175	11.06%
**1d8**	-766.294472	-766.27639	-766.275446	-766.341591	0.2616675	14.57%
**1d9**	-766.292267	-766.274114	-766.27317	-766.339482	1.585065	1.56%
**1d10**	-766.293361	-766.275231	-766.274287	-766.340601	0.8828925	5.11%

E, E', H, G: total energy, total energy with zero point energy (ZPE), enthalpy, and Gibbs free energy

Table S5. ^{13}C NMR calculation details for **1a**

position	1a1	1a2	1a3	1a4	1a5	1a6	1a7	1a8	1a9	1a10	1a11	δ_{calcd}[a]	δ_{corr}[b]	δ_{exp}	Rel err[c]
C-2	157.6974	157.847	157.6885	157.692	157.7295	157.5925	158.9411	159.2487	158.993	159.1597	158.9253	157.94	154.77	157.12	2.3
C-4	81.0439	80.9179	80.7476	81.0601	80.2304	81.0068	80.5784	76.7348	80.5639	76.8973	80.5896	80.68	78.13	76.58	-1.6
C-5	44.9929	45.1351	45.025	44.9899	44.4336	44.9895	45.5389	45.105	45.5078	45.1622	45.5681	45.06	42.80	41.77	-1.0
C-8	14.7106	14.6667	14.6388	14.7147	20.1734	14.7109	11.2104	15.6158	11.2246	15.5794	11.1297	14.48	12.46	13.33	0.9
C-6	24.3041	24.1765	24.2531	24.3066	27.5631	24.2874	29.3635	29.2929	29.4133	29.2414	29.4382	25.46	23.36	24.95	1.6
C-7	13.5019	13.546	13.5944	13.5054	15.3718	13.5574	14.0956	13.5124	14.0895	13.5088	13.9657	13.71	11.70	11.71	0.0
C-1'	185.7112	187.02	187.1561	185.7211	185.0819	186.2543	188.8407	189.3431	189.7939	190.3199	190.6545	186.86	183.46	182.45	-1.0
C-2'	43.9944	41.802	43.4363	43.9972	43.9349	43.1683	44.1279	44.2325	41.921	42.1729	43.6346	43.53	41.28	39.64	-1.6
C-5'	19.9459	14.6849	14.1841	19.9458	19.9843	16.0037	19.9968	19.8976	14.736	14.7237	14.3388	17.40	15.36	15.79	0.4
C-3'	30.3006	30.5551	32.1894	30.298	30.5819	28.4522	30.4739	30.3925	30.7084	30.8749	32.5071	30.23	28.09	28.02	-0.1
C-4'	14.347	9.7405	14.4454	14.3462	14.3065	13.0991	14.2683	14.306	9.8145	9.7855	14.4784	13.66	11.65	11.69	0.0
population	14.99%	4.57%	13.28%	15.07%	5.07%	27.04%	7.83%	3.42%	2.11%	1.23%	5.38%				

[a] Weighted average from the calculated shifts; [b] Obtained by linear fit $\boldsymbol{\delta}_{exp}$ versus $\boldsymbol{\delta}_{calcd}$; [c] $\Delta\boldsymbol{\delta} = \boldsymbol{\delta}_{exp} - \boldsymbol{\delta}_{calcd}$

RMSD = 1.21 ppm.

Table S6. ^{1}H NMR calculation details for **1a**

position	1a1	1a2	1a3	1a4	1a5	1a6	1a7	1a8	1a9	1a10	1a11	δ_{calcd}[a]	δ_{corr}[b]	δ_{exp}	Rel err[c]
H-4	5.6388	5.6491	5.6009	5.6380	5.6666	5.5687	5.7221	5.9380	5.7226	5.9308	5.6816	5.64	5.60	5.55	-0.05
H-5	1.5572	1.5407	1.5003	1.5571	1.7681	1.5160	1.5820	1.5901	1.5770	1.5830	1.5723	1.55	1.57	1.70	0.13
H-8	0.9579	0.9561	0.9450	0.9579	0.9370	0.9434	0.9581	0.9601	0.9497	0.9523	0.9421	0.95	0.97	0.98	0.01
H-6a	1.1460	1.1392	1.1449	1.1456	1.3346	1.1373	1.0134	1.3530	1.0326	1.3453	0.9741	1.14	1.16	1.16	0.00
H-6b	1.6680	1.6579	1.6442	1.6680	1.5935	1.6576	1.5354	1.2252	1.5275	1.2312	1.5096	1.62	1.63	1.53	-0.10
H-7	0.9690	0.9730	0.9664	0.9691	1.0662	0.9658	0.9910	1.1161	0.9905	1.1204	0.9721	0.98	1.00	0.92	-0.08
H-2'	2.7178	3.0784	2.7080	2.7175	2.7028	2.6281	2.7690	2.7540	3.1289	3.1606	2.7493	2.73	2.73	2.87	0.14
H-5'	1.0743	1.0472	1.0128	1.0745	1.0721	1.0573	1.0564	1.0736	1.0373	1.0554	0.9981	1.05	1.08	1.08	0.00
H-3a'	1.3467	2.0076	1.3068	1.3467	1.3510	1.5684	1.3568	1.3608	1.9878	1.9905	1.2967	1.45	1.47	1.53	0.06
H-3b'	1.8505	1.5898	1.8971	1.8503	1.8602	1.5273	1.8338	1.8734	1.6017	1.6262	1.9144	1.75	1.76	1.66	-0.10
H-4'	0.8889	0.8262	1.0615	0.8889	0.8743	0.9952	0.8754	0.8998	0.8238	0.8447	1.0574	0.94	0.97	0.95	-0.02
population	14.99%	4.57%	13.28%	15.07%	5.07%	27.04%	7.83%	3.42%	2.11%	1.23%	5.38%				

RMSD = 0.08 ppm.

Table S7. ^{13}C NMR calculation details for **1b**

position	1b1	1b2	1b3	1b4	1b5	1b6	1b7	1b8	1b9	1b10	1b11	1b12	δ_{calcd}
C-2	159.142	158.8523	159.0056	158.7956	158.9038	158.5735	159.0914	159.1443	158.8605	158.671	159.6656	159.6054	158.94
C-4	75.8383	80.2142	75.9886	80.277	76.0088	80.3281	75.7709	75.8457	80.2137	80.0873	79.8239	80.1166	78.85
C-5	44.7983	45.4359	44.8243	45.5317	44.6172	45.3158	44.76	44.7986	45.4391	45.394	45.2945	45.3476	45.21
C-8	15.2613	11.0313	15.2021	11.1028	15.0779	11.0298	15.2761	15.261	11.031	11.1008	16.0849	16.108	12.70
C-6	29.1447	28.1547	29.0424	28.2219	29.1124	28.2937	29.0192	29.1452	28.1543	28.137	24.1186	24.147	28.19
C-7	13.4964	14.0611	13.3919	14.0776	13.3275	14.0753	13.3913	13.4959	14.0609	14.0655	13.6022	13.597	13.84
C-1'	187.4162	187.0725	188.3943	187.9661	188.9167	188.3185	187.9669	187.4317	187.1021	187.4699	189.2422	190.255	187.66
C-2'	44.229	44.0154	42.0898	42.2465	43.5151	43.5623	43.1533	44.2282	44.0131	43.0757	44.2739	42.2172	43.77
C-5'	19.9382	19.8905	14.7456	14.6061	14.2473	14.3721	16.1021	19.942	19.8917	16.1425	20.0347	14.7154	18.31
C-3'	30.4742	30.4502	30.7709	30.914	32.4502	32.3443	28.6609	30.4747	30.4586	28.732	30.5734	30.4946	30.65
C-4'	14.2815	14.3271	9.8604	9.7807	14.395	14.4671	12.9024	14.2819	14.3261	13.0122	14.337	9.8933	13.84
population	10.07%	21.23%	2.94%	4.13%	5.83%	9.37%	2.27%	10.06%	21.23%	5.89%	5.45%	1.52%	

Table S8. ^{1}H NMR calculation details for **1b**

position	1b1	1b2	1b3	1b4	1b5	1b6	1b7	1b8	1b9	1b10	1b11	1b12	δ_{calcd}
H-4	6.0131	5.6707	6.0066	5.6769	5.9457	5.6270	5.9450	6.0131	5.6708	5.6205	5.7379	5.7362	5.77
H-5	1.6449	1.5487	1.6268	1.5362	1.5892	1.4960	1.6054	1.6444	1.5486	1.5062	1.6272	1.6315	1.57
H-8	0.9777	0.9668	0.9682	0.9594	0.9574	0.9443	0.9729	0.9777	0.9669	0.9624	0.9172	0.9177	0.96
H-6a	1.2193	1.5209	1.2246	1.5279	1.2185	1.5233	1.2262	1.2193	1.5209	1.5032	1.6209	1.6166	1.43
H-6b	1.4909	1.0524	1.4910	1.0531	1.4834	1.0597	1.4915	1.4908	1.0523	1.0489	1.0691	1.0696	1.19
H-7	1.0481	1.0154	1.0625	1.0214	1.0498	1.0071	1.0357	1.0481	1.0154	1.0125	0.9919	0.9889	1.02
H-2'	2.7458	2.7385	3.1071	3.1035	2.7641	2.7433	2.6973	2.7458	2.7391	2.6675	2.7782	3.1256	2.77
H-5'	1.0858	1.0728	1.0617	1.0526	1.0278	1.0185	1.0641	1.0858	1.0727	1.0586	1.0614	1.0317	1.06
H-3a'	1.3826	1.3762	2.0389	2.0331	1.3256	1.3094	1.6040	1.3827	1.3766	1.5954	1.3781	2.0319	1.44
H-3b'	1.8731	1.8740	1.6073	1.6223	1.9170	1.9278	1.5352	1.8735	1.8740	1.5384	1.8436	1.6067	1.83
H-4'	0.8969	0.8957	0.8287	0.8230	1.0632	1.0662	0.9982	0.8970	0.8957	0.9935	0.8870	0.8280	0.92
population	10.07%	21.23%	2.94%	4.13%	5.83%	9.37%	2.27%	10.06%	21.23%	5.89%	5.45%	1.52%	

Table S9. ^{13}C NMR calculation details for **1c**

position	1c1	1c2	1c3	1c4	1c5	1c6	1c7	1c8	1c9	1c10	1c11	δ_{calcd}^{a}	δ_{corr}^{b}	δ_{exp}	Rel err^{c}
C-2	158.0317	157.8739	158.0516	158.2118	157.981	157.7878	158.0237	157.8501	157.5636	157.5457	159.0533	157.88	154.70	156.96	2.3
C-4	76.4889	81.0024	76.4565	76.5655	75.9717	81.112	76.3383	80.9738	80.5332	81.1573	80.2472	79.50	76.90	76.78	-0.1
C-5	44.9997	45.2239	44.6439	44.9857	44.5993	44.8905	44.8253	45.2647	44.8848	44.8998	45.3146	44.99	42.64	41.44	-1.2
C-8	15.2652	11.1166	15.1084	15.2991	15.1987	11.0434	15.1705	11.1503	11.071	11.0108	16.1721	12.59	10.48	13.12	2.6
C-6	29.0584	28.2512	29.0359	29.0855	29.0309	28.1953	29.0172	28.2119	28.1806	28.1064	24.188	28.21	25.99	24.97	-1.0
C-7	13.4596	14.1095	13.4648	13.4626	13.4856	14.2083	13.5098	14.1311	14.1612	14.1921	13.6414	13.93	11.81	12.06	0.2
C-1'	187.0554	186.9477	184.5299	188.9152	187.8637	184.3705	185.883	187.8974	186.8529	185.0963	190.5055	186.91	183.52	182.20	-1.3
C-2'	43.5512	43.498	41.3059	43.3945	42.2	41.5038	42.8825	43.364	42.4535	42.8965	43.7207	42.91	40.58	39.58	-1.0
C-5'	22.5166	22.4471	15.8761	18.5575	22.1147	15.8669	15.2735	18.4715	22.1404	15.172	22.8493	19.95	17.79	17.65	-0.1
C-3'	29.0651	28.9573	28.2657	27.4401	32.252	28.2558	29.3463	27.2906	31.7692	29.3449	29.0459	29.63	27.41	27.02	-0.4
C-4'	14.5959	14.5624	9.8013	12.9445	14.6143	9.8227	13.965	13.0649	14.5873	13.978	14.4993	13.92	11.81	11.84	0.0
population	6.66%	18.21%	2.38%	2.49%	10.24%	4.36%	7.61%	11.34%	18.64%	12.37%	5.70%				

^{a} Weighted average from the calculated shifts; ^{b} Obtained by linear fit $\boldsymbol{\delta}_{exp}$ versus $\boldsymbol{\delta}_{calcd}$; ^{c} $\Delta\boldsymbol{\delta} = \boldsymbol{\delta}_{exp} - \boldsymbol{\delta}_{calcd}$

RMSD = 1.26 ppm.

Table S10. ^{1}H NMR calculation details for **1c**

position	1c1	1c2	1c3	1c4	1c5	1c6	1c7	1c8	1c9	1c10	1c11	δ_{calcd}^{a}	δ_{corr}^{b}	δ_{exp}	Rel err^{c}
H-4	5.9736	5.6350	5.9731	5.9380	6.0548	5.6407	5.9434	5.5993	5.6991	5.5751	5.7345	5.75	5.60	5.55	-0.05
H-5	1.6284	1.5401	1.6215	1.6003	1.6261	1.5653	1.6077	1.5137	1.5600	1.5644	1.5863	1.57	1.59	1.74	0.15
H-8	0.9717	0.9635	0.9688	0.9822	0.9771	0.9520	0.9719	0.9637	0.9589	0.9548	0.9126	0.96	1.01	0.95	-0.06
H-6a	1.4862	1.0457	1.4707	1.4751	1.4674	1.0269	1.4601	1.0361	1.0343	1.0137	1.0813	1.16	1.20	1.20	0.00
H-6b	1.2135	1.5072	1.2124	1.2114	1.2254	1.4984	1.2064	1.4854	1.4920	1.4727	1.6534	1.42	1.45	1.51	0.06
H-7	1.0654	1.0129	1.0575	1.0527	1.0576	1.0027	1.0405	1.0160	1.0122	1.0074	0.9923	1.02	1.07	0.97	-0.10
H-2'	2.7607	2.7782	3.0283	2.6571	2.9907	3.0190	2.6880	2.6366	2.9658	2.6724	2.8653	2.82	2.79	2.87	0.08
H-5'	1.2318	1.2371	1.1295	1.2346	1.1862	1.1386	1.1115	1.2378	1.1868	1.1063	1.2307	1.19	1.23	1.18	-0.05
H-3a'	1.2775	1.2768	1.8187	1.4005	1.4196	1.8405	1.1486	1.3904	1.4051	1.1458	1.2751	1.34	1.38	1.46	0.08
H-3b'	1.8376	1.8418	1.5215	1.5605	1.7254	1.5140	1.6262	1.5525	1.7262	1.6361	1.8283	1.70	1.72	1.65	-0.07
H-4'	0.8587	0.8474	0.8268	0.9729	0.8583	0.8272	1.0279	0.9706	0.8539	1.0216	0.8194	0.90	0.95	0.91	-0.04
population	6.66%	18.21%	2.38%	2.49%	10.24%	4.36%	7.61%	11.34%	18.64%	12.37%	5.70%				

RMSD = 0.08 ppm.

Table S11. ^{13}C NMR calculation details for **1d**

position	1d1	1d2	1d3	1d4	1d5	1d6	1d7	1d8	1d9	1d10	δ_{calcd}
C-2	158.5461	158.5285	158.5013	158.5445	158.7182	159.1195	159.5593	159.7103	159.1223	159.4508	158.90
C-4	80.0505	80.0754	80.0871	80.0564	80.1262	79.2774	80.0924	76.0856	79.2772	77.4499	79.33
C-5	45.1223	45.1464	45.0522	45.1206	45.07	44.8885	45.6918	45.5635	44.886	41.9112	45.07
C-8	14.5811	14.6628	14.6389	14.5814	14.6008	19.8366	11.2869	15.5153	19.8357	15.5955	14.58
C-6	24.2763	24.2585	24.157	24.2748	24.1934	26.977	29.2863	29.1881	26.9751	27.0669	25.75
C-7	13.4563	13.4791	13.4367	13.4547	13.4193	15.7483	13.9549	13.6022	15.7492	8.0149	13.32
C-1'	188.112	185.1593	188.9162	188.1177	186.6962	187.8457	190.441	190.7354	187.8374	190.4062	188.74
C-2'	43.0152	41.4734	43.3061	43.0168	43.1346	43.1661	43.5683	43.7351	43.1684	43.6516	43.22
C-5'	22.7835	15.913	18.7259	22.7827	15.2024	22.2194	23.094	23.0764	22.217	22.9393	21.63
C-3'	29.5594	28.2116	27.334	29.5597	29.4414	29.2895	29.2301	29.117	29.2934	29.1879	29.17
C-4'	14.5581	9.6921	12.7599	14.557	13.973	14.521	14.4872	14.4915	14.5221	14.5792	14.18
population	22.52%	2.66%	9.87%	22.66%	8.43%	1.56%	11.06%	14.57%	1.56%	5.11%	

Table S12. ^{1}H NMR calculation details for **1d**

position	1d1	1d2	1d3	1d4	1d5	1d6	1d7	1d8	1d9	1d10	δ_{calcd}
H-4	5.6925	5.6719	5.6419	5.6921	5.6365	5.7218	5.7417	5.9571	5.7219	5.6004	5.72
H-5	1.5237	1.5037	1.4953	1.5236	1.5107	1.7037	1.6299	1.6318	1.7038	1.9513	1.57
H-8	0.9676	0.9637	0.9576	0.9675	0.9519	0.9465	0.9415	0.9570	0.9465	0.9368	0.96
H-6a	1.1506	1.1286	1.1355	1.1505	1.1354	1.3363	0.9821	1.3441	1.3362	1.6584	1.19
H-6b	1.6594	1.6480	1.6668	1.6594	1.6716	1.5666	1.5134	1.1723	1.5665	1.5411	1.56
H-7	0.9780	0.9739	0.9682	0.9779	0.9628	1.0970	0.9798	1.1216	1.0969	0.8951	1.00
H-2'	2.9129	3.0308	2.6722	2.9126	2.6816	2.8626	2.9361	2.9275	2.8625	2.8954	2.87
H-5'	1.2315	1.1451	1.2447	1.2317	1.1150	1.2112	1.2349	1.2614	1.2111	1.2544	1.23
H-3a'	1.8473	1.5273	1.5439	1.8473	1.6346	1.8466	1.8301	1.8512	1.8472	1.8508	1.79
H-3b'	1.3052	1.8064	1.4130	1.3051	1.1188	1.3005	1.3043	1.3161	1.3004	1.2998	1.31
H-4'	0.8200	0.8180	0.9658	0.8201	1.0219	0.8256	0.8108	0.8368	0.8255	0.8458	0.85
population	22.52%	2.66%	9.87%	22.66%	8.43%	1.56%	11.06%	14.57%	1.56%	5.11%	

Figure S21. Left: Regression analysis of experimental versus calculated ^{13}C NMR chemical shifts of **1** at mPW1PW91/6-311g(d,p) level; linear fitting was shown as a line; Right: Relative chemical shift errors between scaled ^{13}C NMR and experimental ^{13}C NMR for **1** (δ_{corr} obtained by linear fit δ_{exp} versus δ_{calcd}).

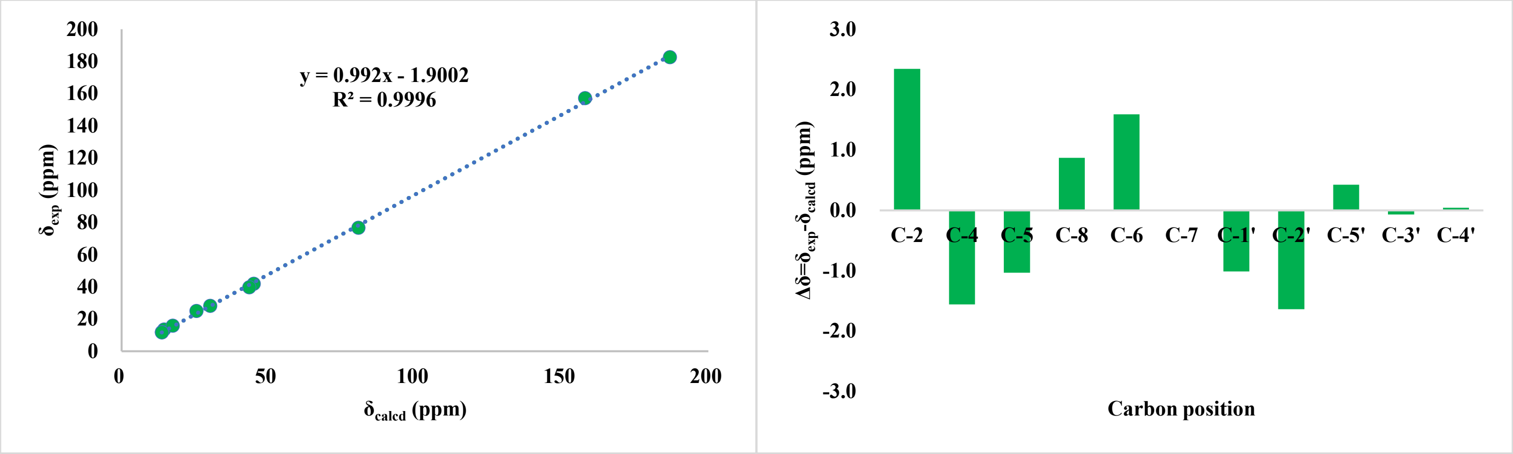

Figure S22. Left: Regression analysis of experimental versus calculated ^{1}H NMR chemical shifts of **1** at mPW1PW91/6-311g(d,p) level; linear fitting was shown as a line; Right: Relative chemical shift errors between scaled ^{1}H NMR and experimental ^{1}H NMR for **1** (δ_{corr} obtained by linear fit δ_{exp} versus δ_{calcd}).

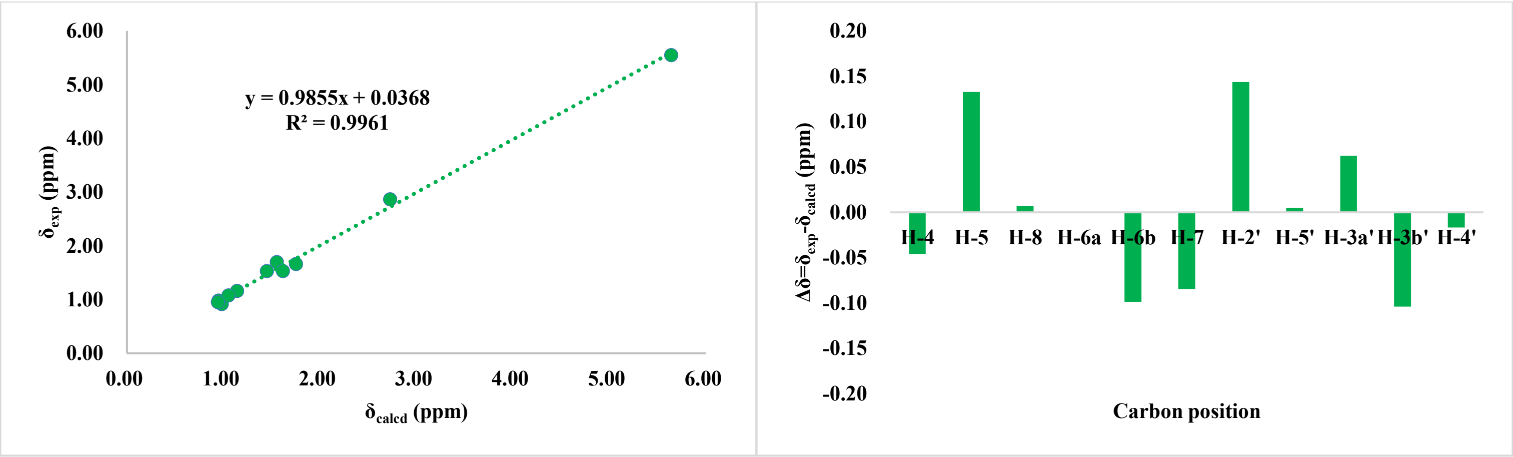

Figure S23. Left: Regression analysis of experimental versus calculated ^{13}C NMR chemical shifts of **2** at mPW1PW91/6-311g(d,p) level; linear fitting was shown as a line; Right: Relative chemical shift errors between scaled ^{13}C NMR and experimental ^{13}C NMR for **2** (δ_{corr} obtained by linear fit δ_{exp} versus δ_{calcd}).

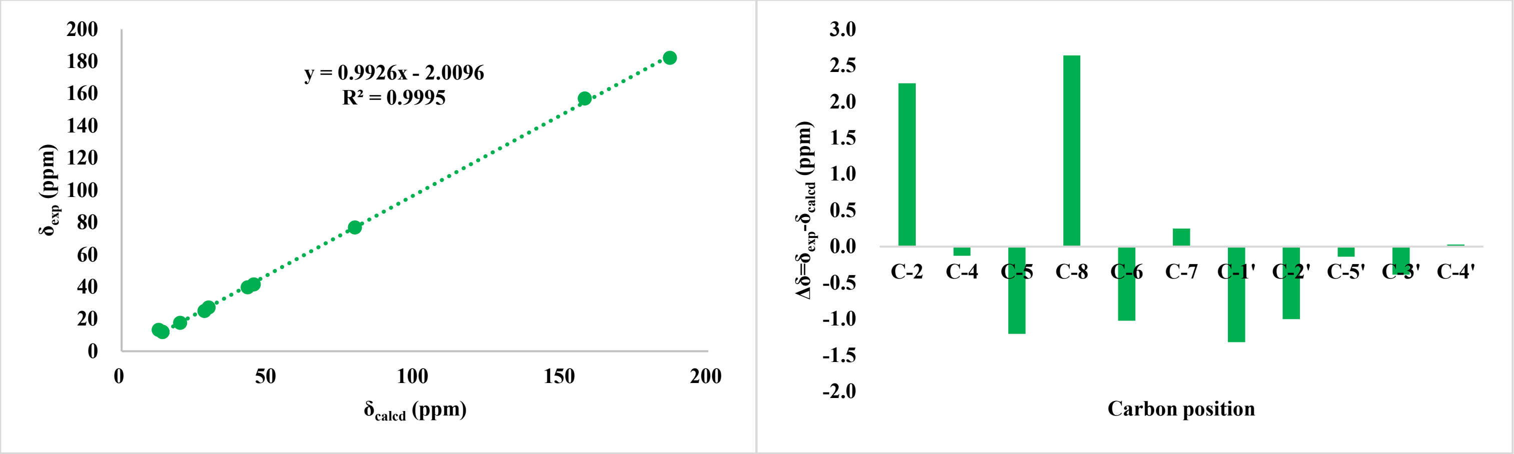

Figure S24. Left: Regression analysis of experimental versus calculated ^{1}H NMR chemical shifts of **2** at mPW1PW91/6-311g(d,p) level; linear fitting was shown as a line; Right: Relative chemical shift errors between scaled ^{1}H NMR and experimental ^{1}H NMR for **2** (δ_{corr} obtained by linear fit δ_{exp} versus δ_{calcd}).

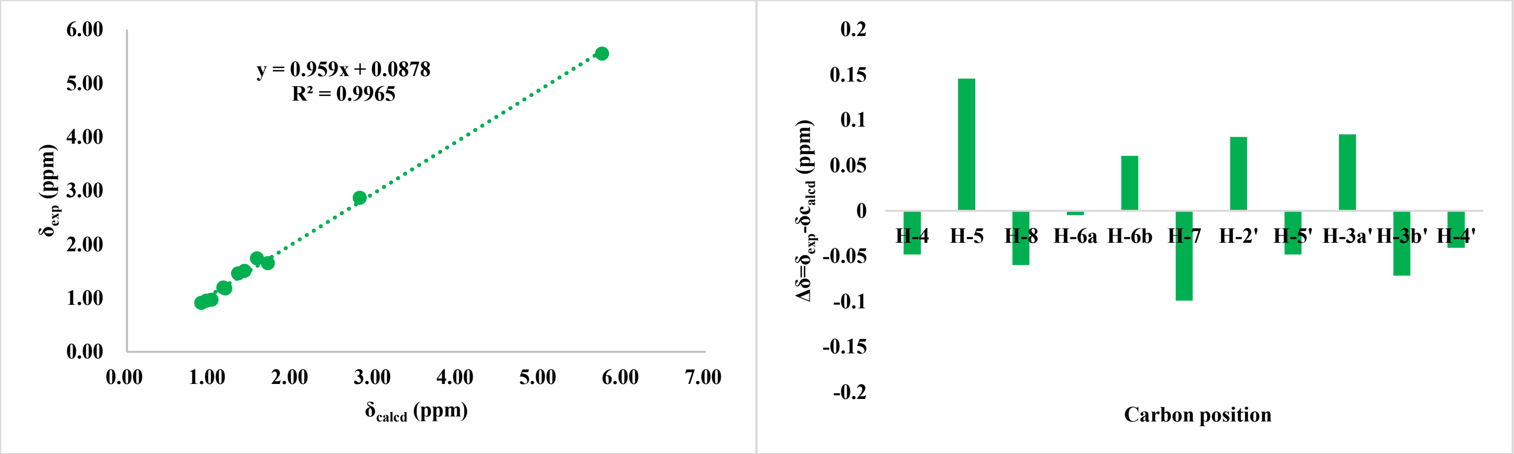

Table S13. The DP4+ probabilities of **1a**, **1b**, **1c** and **1d**.[5-7]

Functional		Solvent		Basis Set		Tape of Data	Functional		Solvent		Basis Set		Tape of Data
mPW1PW91		PCM		6-311G(d,p)		Unscaled Shifts	mPW1PW91		PCM		6-311G(d,p)		Unscaled Shifts
		DP4+	97.64%	0.01%	2.35%	0.00%			DP4+	0.18%	0.00%	99.79%	0.04%
Nuclei	sp2?	Experiental (**1**)	**1a**	**1b**	**1c**	**1d**	Nuclei	sp2?	Experiental (**2**)	**1a**	**1b**	**1c**	**1d**
C	×	157.12	157.9	158.9	157.9	158.9	C	×	156.96	157.9	158.9	157.9	158.9
C		76.58	80.7	78.8	79.5	79.3	C		76.78	80.7	78.8	79.5	79.3
C		41.77	45.1	45.2	45.0	45.1	C		41.44	45.1	45.2	45.0	45.1
C		13.33	14.5	12.7	12.6	14.6	C		13.12	14.5	12.7	12.6	14.6
C		24.95	25.5	28.2	28.2	25.8	C		24.97	25.5	28.2	28.2	25.8
C		11.71	13.7	13.8	13.9	13.3	C		12.06	13.7	13.8	13.9	13.3
C	×	182.45	186.9	187.7	186.9	188.7	C	×	182.20	186.9	187.7	186.9	188.7
C		39.64	43.5	43.8	42.9	43.2	C		39.58	43.5	43.8	42.9	43.2
C		15.79	17.4	18.3	19.9	21.6	C		17.65	17.4	18.3	19.9	21.6
C		28.02	30.2	30.6	29.6	29.2	C		27.02	30.2	30.6	29.6	29.2
C		11.69	13.7	13.8	13.9	14.2	C		11.84	13.7	13.8	13.9	14.2
H		5.55	5.64	5.77	5.75	5.72	H		5.55	5.64	5.77	5.75	5.72
H		1.70	1.55	1.57	1.57	1.57	H		1.74	1.55	1.57	1.57	1.57
H		0.98	0.95	0.96	0.96	0.96	H		0.95	0.95	0.96	0.96	0.96
H		1.16	1.14	1.43	1.16	1.19	H		1.20	1.14	1.43	1.16	1.19
H		1.53	1.62	1.19	1.42	1.56	H		1.51	1.62	1.19	1.42	1.56
H		0.92	0.98	1.02	1.02	1.00	H		0.97	0.98	1.02	1.02	1.00
H		2.87	2.73	2.77	2.82	2.87	H		2.87	2.73	2.77	2.82	2.87
H		1.08	1.05	1.06	1.19	1.23	H		1.18	1.05	1.06	1.19	1.23
H		1.53	1.45	1.44	1.34	1.79	H		1.46	1.45	1.44	1.34	1.79
H		1.66	1.75	1.83	1.70	1.31	H		1.65	1.75	1.83	1.70	1.31
H		0.95	0.94	0.92	0.90	0.85	H		0.91	0.94	0.92	0.90	0.85

2.2 ECD calculation details for **1a**, **1c, 1e** and **1f**

Conformation searches based on molecular mechanics with MMFF94s force field were performed for (4*R*,5*S*,2′*S*)-**1** (**1a**), (4*R*,5*R*,2′*R*)-**1** (**1c**), (4*S*,5*S*,2′*S*)-**1** (**1e**) and (4*S*,5*R*,2′*R*)-**1** (**1f**) gave 15, 16, 16 and 15 conformers with populations higher than 1%. All these conformers were further optimized by the density functional theory method at the B3LYP/6-31G(d) level in Gaussian 09 program package, led to eleven (**1a1** to **1a11**), eleven (**1c1** to **1c11**), eleven (**1e1** to **1e11**) and eleven (**1f1** to **1f11**) conformers within a 2.0 kcal/mol energy threshold from global minimum, respectively. These predominant conformers were subjected to theoretical calculation of ECD using time-dependent density functional theory (TDDFT) at CAM-B3LYP/6-311+G (d,p) level in air. The calculated ECD curves for (4*R*,5*S*,2′*S*)-**1** (**1a**), (4*R*,5*R*,2′*R*)-**1** (**1c**), (4*S*,5*S*,2′*S*)-**1** (**1e**), (4*S*,5*R*,2′*R*)-**1** (**1f**) and their and weighted ECD were all generated using SpecDis 1.60 with σ = 0.3 eV, and UV shift 2, respectively.[8] As shown in **Figure S27**, the calculated ECD spectrum of (4*R*,5*S*,2′*S*)-**1** (**1a**) matched well with the **1** of the experimental one. Meanwhile, the calculated ECD spectrum of (4*S*,5*S*,2′*S*)-**1** (**1e**) matched well with **2** of the experimental one, suggesting the absolute configuration of **1** to be 4*R*,5*S*,2′*S*, and that of **2** to be 4*S*,5*S*,2′*S*.

Figure S25. Optimized conformers of **1e** (**1e1**-**1e11**).

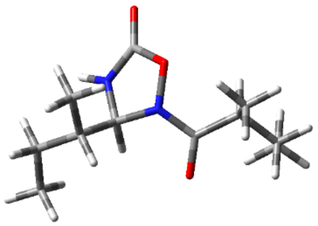

1e1

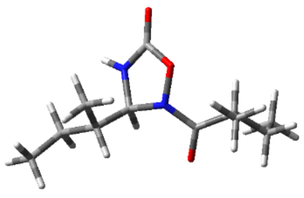

1e2

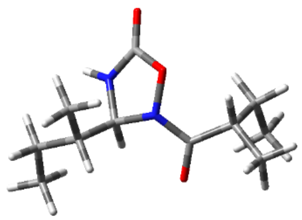

1e3

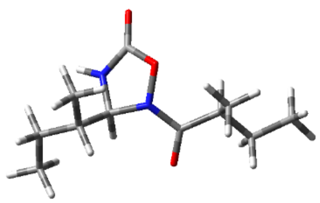

1e4

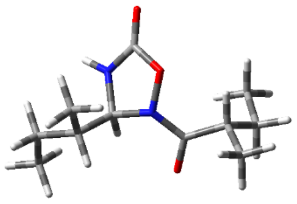

1e5

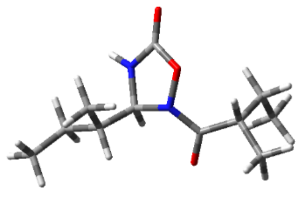

1e6

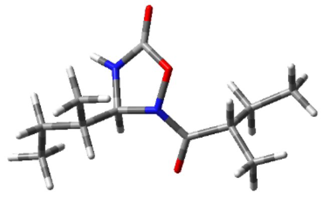

1e7

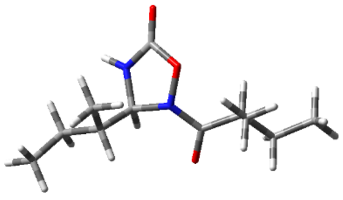

1e8

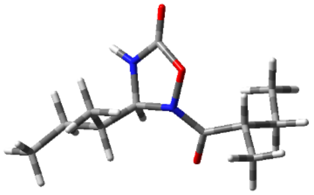 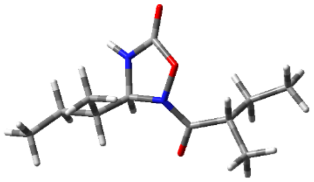 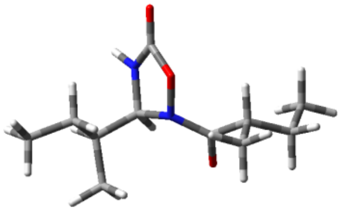

1e9 **1e10** **1e11**

Table S14. Energy analysis for conformers of **1e1-1e11** at B3LYP/6-31G(d) level in the gas phase

species	*E'=E+ZPE*	*E*	*H*	*G*	*ΔE* (kcal/mol)	P_E%
**1e1**	-766.294116	-766.276012	-766.275068	-766.341056	0.711585	6.90%
**1e2**	-766.294803	-766.276598	-766.275653	-766.342177	0.0081575	22.62%
**1e3**	-766.292372	-766.274166	-766.273222	-766.33966	1.587575	1.57%
**1e4**	-766.293105	-766.274954	-766.27401	-766.34013	1.29265	2.59%
**1e5**	-766.294115	-766.276012	-766.275068	-766.341059	0.7097025	6.92%
**1e6**	-766.292877	-766.274704	-766.27376	-766.339932	1.416895	2.10%
**1e7**	-766.293419	-766.275217	-766.274273	-766.340717	0.9243075	4.82%
**1e8**	-766.293941	-766.275618	-766.274674	-766.341499	0.4336025	11.03%
**1e9**	-766.294806	-766.276599	-766.275655	-766.34219	0	22.93%
**1e10**	-766.294112	-766.275842	-766.274898	-766.34161	0.36395	12.41%
**1e11**	-766.294108	-766.276008	-766.275064	-766.340942	0.78312	6.11%

E, *E'*, *H*, *G*: total energy, total energy with zero point energy (ZPE), enthalpy, and Gibbs free energy

Figure S26. Optimized conformers of **1f** (**1f1-1f11**).

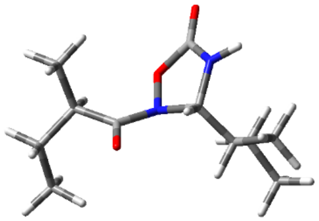 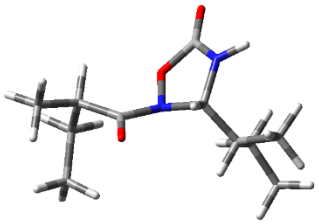 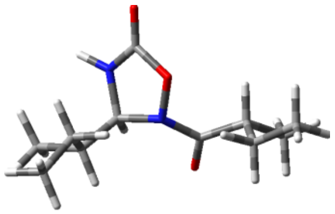 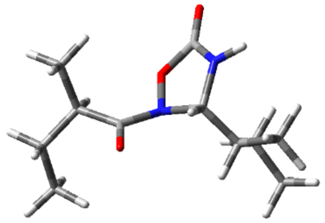

1f1 **1f2** **1f3** **1f4**

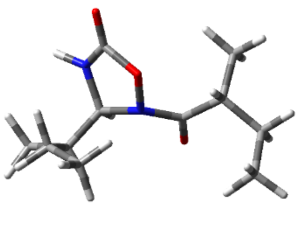

1f5

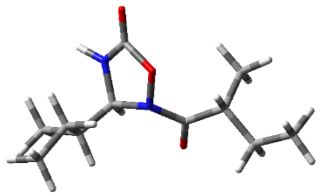

1f6

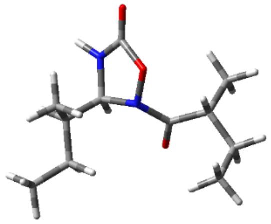

1f7

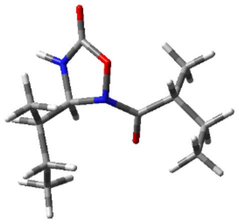

1f8

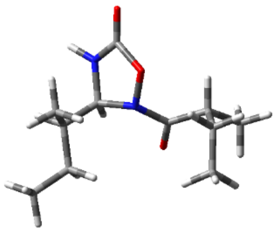

1f9

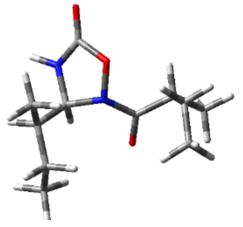

1f10

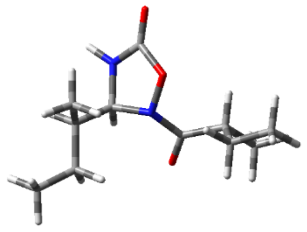

1f11

Table S15. Energy analysis for conformers of **1f1-1f11** at B3LYP/6-31G(d) level in the gas phase

species	*E'*=*E*+*ZPE*	*E*	*H*	*G*	*ΔE* (kcal/mol)	P_E%
**1f1**	-766.294853	-766.276636	-766.275692	-766.341877	0	21.21%
**1f2**	-766.293322	-766.275185	-766.274241	-766.340223	1.037885	3.68%
**1f3**	-766.293927	-766.275694	-766.27475	-766.341216	0.4147775	10.53%
**1f4**	-766.294853	-766.276638	-766.275694	-766.341875	0.001255	21.17%
**1f5**	-766.29267	-766.274455	-766.273511	-766.340206	1.0485525	3.61%
**1f6**	-766.293689	-766.275333	-766.274389	-766.3413	0.3620675	11.51%
**1f7**	-766.294663	-766.27655	-766.275606	-766.34148	0.2491175	13.93%
**1f8**	-766.294491	-766.276534	-766.27559	-766.340521	0.85089	5.04%
**1f9**	-766.293111	-766.275105	-766.27416	-766.339588	1.4363475	1.88%
**1f10**	-766.293096	-766.275124	-766.274179	-766.339562	1.4526625	1.83%
**1f11**	-766.293704	-766.275618	-766.274674	-766.340621	0.78814	5.61%

E, *E'*, *H*, *G*: total energy, total energy with zero point energy (ZPE), enthalpy, and Gibbs free energy

Figure S27. Comparison of the calculated ECD spectra with the experimental spectrum.

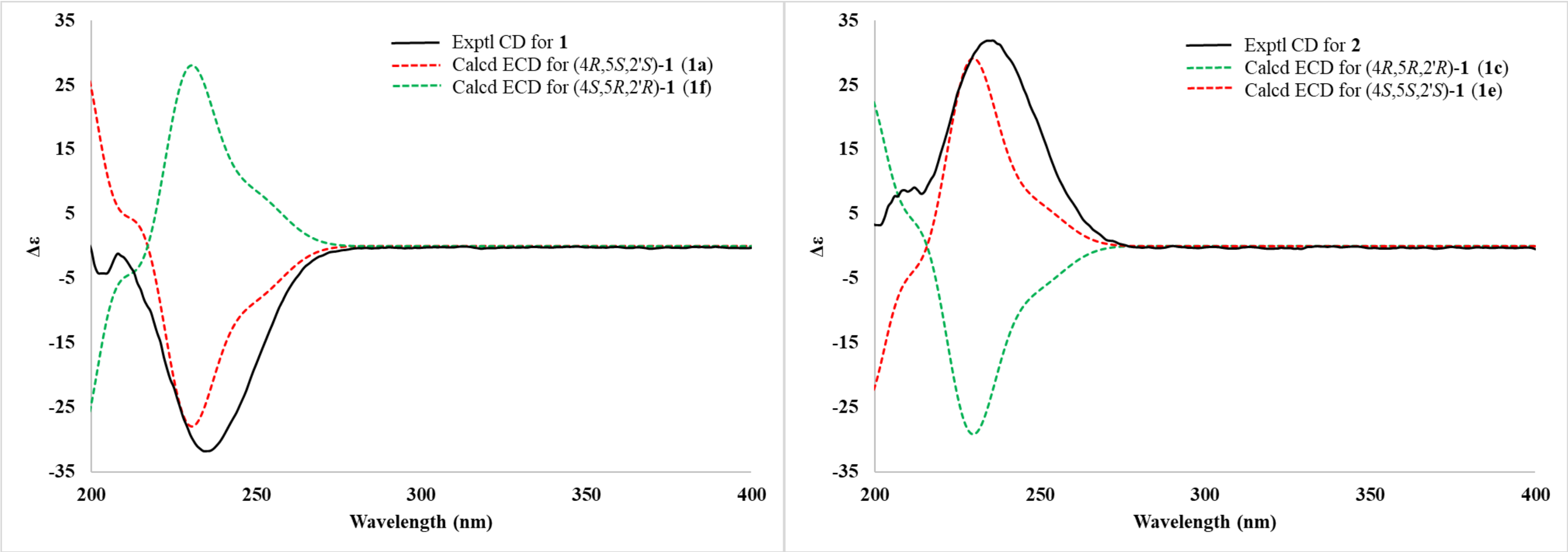

2.3 Determination of the substitution pattern.

The ^{13}C NMR chemical shifts have been calculated using the GIAO method (the same as above) on the most stable conformer for each regioisomer (see table below) and the DP4+ probability was calculated.[6-7]

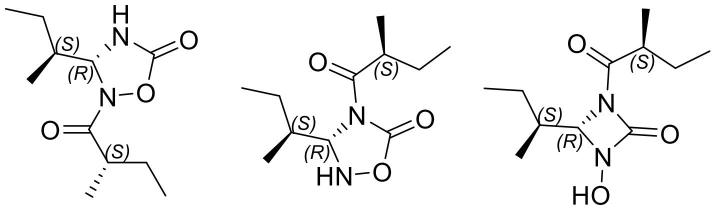

1a **1g** **1h**

Figure S28. Optimized conformers of **1g** (**1g1-1g10**).

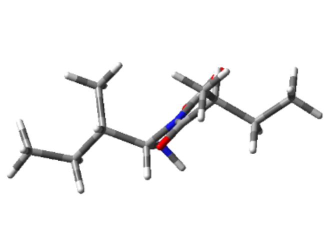

1g1

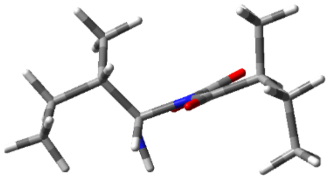

1g2

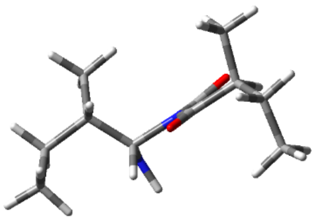

1g3

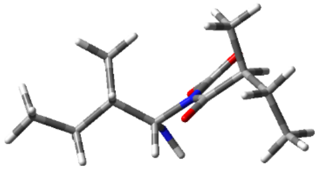

1g4

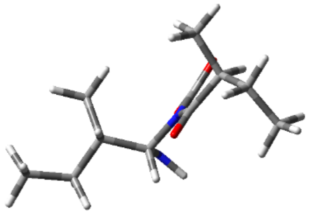

1g5

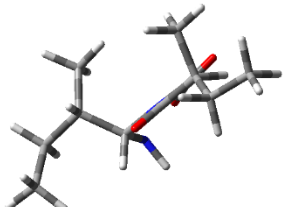

1g6

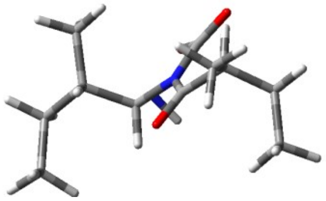

1g7

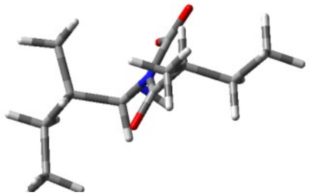

1g8

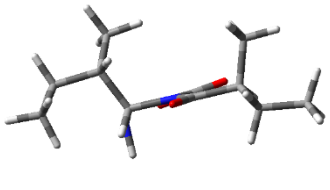

1g9

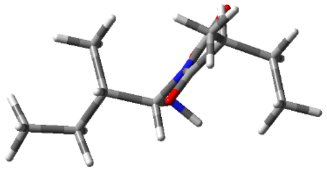

1g10

Table S16. Energy analysis for conformers of **1g1-1g10** at B3LYP/6-31G(d) level in the gas phase

species	*E'*=*E*+*ZPE*	*E*	*H*	*G*	*ΔE* (kcal/mol)	P_E%
**1g1**	-766.291066	-766.273223	-766.272279	-766.337314	0.1123225	12.55%
**1g2**	-766.291066	-766.273223	-766.272279	-766.337319	0.109185	12.62%
**1g3**	-766.291384	-766.273565	-766.272621	-766.337493	0	15.17%
**1g4**	-766.291383	-766.273565	-766.27262	-766.337492	0.0006275	15.16%
**1g5**	-766.290088	-766.272151	-766.271207	-766.336879	0.385285	7.92%
**1g6**	-766.289583	-766.271771	-766.270827	-766.335809	1.05671	2.55%
**1g7**	-766.290228	-766.272286	-766.271342	-766.337088	0.2541375	9.88%
**1g8**	-766.29009	-766.272152	-766.271208	-766.336885	0.38152	7.97%
**1g9**	-766.28978	-766.27205	-766.271106	-766.335639	1.163385	2.13%
**1g10**	-766.290587	-766.27264	-766.271696	-766.33742	0.0458075	14.05%

E, *E'*, *H*, *G*: total energy, total energy with zero point energy (ZPE), enthalpy, and Gibbs free energy

Figure S29. Optimized conformers of **1h** (**1h1**-**1h3**).

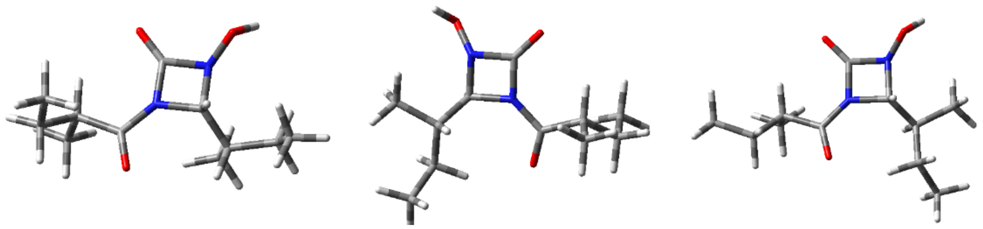

1h1 **1h2** **1h3**

Table S17. Energy analysis for conformers of **1h1**-**1h3** at B3LYP/6-31G(d) level in the gas phase

species	*E'*=*E*+*ZPE*	*E*	*H*	*G*	*ΔE* (kcal/mol)	P_E%
**1h1**	-766.268634	-766.249848	-766.248904	-766.31705	1.037885	14.04%
**1h2**	-766.267439	-766.248639	-766.247695	-766.316072	1.65158	4.98%
**1h3**	-766.270367	-766.251592	-766.250647	-766.318704	0	80.97%

E, *E'*, *H*, *G*: total energy, total energy with zero point energy (ZPE), enthalpy, and Gibbs free energy

Table S18. ^{13}C NMR calculation details for **1g**

**position**	**1g1**	**1g2**	**1g3**	**1g4**	**1g5**	**1g6**	**1g7**	**1g8**	**1g9**	**1g10**	**δ_{calcd}**
C-2	162.6432	162.6437	162.6067	162.6061	162.7075	162.6749	162.698	162.708	162.5493	162.5605	162.63
C-4	78.7623	78.7631	83.4697	83.478	78.6391	78.9161	78.8983	78.64	83.8677	83.7732	81.00
C-1'	184.4082	184.4098	183.8726	183.8731	185.3701	183.236	185.1699	185.3714	183.147	185.1679	184.52
C-2'	44.6801	44.6784	45.0754	45.0734	44.0415	40.9575	43.0767	44.0414	41.415	43.7087	44.24
C-5'	21.1577	21.1597	21.2176	21.2187	17.3521	14.5622	13.9444	17.3568	14.6054	13.9319	18.54
C-3'	29.447	29.4466	29.1856	29.1865	27.6307	29.3639	31.4623	27.6364	29.5349	31.7043	29.60
C-4'	13.7621	13.7624	13.7338	13.7351	12.3401	8.9472	13.6209	12.3415	8.8898	13.6888	13.28
C-5	41.4266	41.4325	41.2985	41.294	41.7149	40.8774	41.4695	41.7155	41.149	41.1744	41.38
C-6	28.6803	28.6802	28.8646	28.8648	28.7396	29.0568	28.7156	28.7367	28.9507	29.0292	28.81
C-8	14.7224	14.7226	10.1347	10.1331	14.9526	14.8351	14.8132	14.9514	10.0322	10.2619	12.65
C-7	12.702	12.704	13.2159	13.2156	12.6201	12.5894	12.5331	12.6207	13.2699	13.1765	12.90
population	12.55%	12.62%	15.17%	15.16%	7.92%	2.55%	9.88%	7.97%	2.13%	14.05%	

Table S19. ^{13}C NMR calculation details for **1h**

position	1h1	1h2	1h3	δ_{calcd}
C-2	89.1319	89.098	83.7643	84.78
C-4	162.4175	162.2763	161.9614	162.04
C-5	43.8431	44.3136	39.0006	39.95
C-6	28.7883	28.5652	29.5766	29.42
C-8	15.3135	15.3214	13.8436	14.12
C-7	12.849	12.7867	12.9206	12.90
C-1'	183.0277	183.0198	182.797	182.84
C-2'	46.8253	46.4001	46.7224	46.72
C-5'	21.074	17.5277	20.9669	20.81
C-3'	30.3019	27.268	30.8867	30.62
C-4'	13.7008	12.3817	13.7383	13.67
population	14.04%	4.98%	80.97%	

Table S20. The DP4+ probabilities of **1a**, **1g** and **1h**.

Functional		Solvent	Basis Set		Tape of Data
mPW1PW91		PCM	6-311G(d,p)		Unscaled Shifts
		DP4+	99.92%	0.08%	0.00%
Nuclei	sp2?	Experiental (**1**)	**1a**	**1g**	**1h**
C	×	157.12	157.94	162.63	162.04
C		76.58	80.68	81.00	84.78
C		41.77	45.06	41.38	39.95
C		13.33	14.48	12.65	14.12
C		24.95	25.46	28.81	29.42
C		11.71	13.71	12.90	12.90
C	×	182.45	186.86	184.52	182.84
C		39.64	43.53	44.24	46.72
C		15.79	17.40	18.54	20.81
C		28.02	30.23	29.60	30.62
C		11.69	13.66	13.28	13.67

3. References

1 H. Goto and E. Osawa, *J. Am. Chem. Soc*, 1989, **111**, 8950-8951.

2 H. Gotō and E. Ōsawa, *J. Chem. Soc., Perkin Trans. 2*, 1993, 187-198.

3 M.J. Frisch, G.W. Trucks, H.B. Schlegel, G.E. Scuseria, M.A. Robb, J.R. Cheeseman, G. Scalmani, V. Barone, B. Mennucci, G.A. Petersson, H. Nakatsuji, M. Caricato, X. Li, H.P. Hratchian, A.F. Izmaylov, J. Bloino, G. Zheng, J.L. Sonnenberg, M. Hada, M. Ehara, K. Toyota, R. Fukuda, J. Hasegawa, M. Ishida, T. Nakajima, Y. Honda, O. Kitao, H. Nakai, T. Vreven, J.A. Montgomery, Jr., J. E. Peralta, F. Ogliaro, M. Bearpark, J.J. Heyd, E. Brothers, K.N. Kudin, V.N. Staroverov, R. Kobayashi, J. Normand, K. Raghavachari, A. Rendell, J.C. Burant, S.S. Iyengar, J. Tomasi, M. Cossi, N. Rega, J.M. Millam, M. Klene, J.E. Knox, J.B. Cross, V. Bakken, C. Adamo, J. Jaramillo, R. Gomperts, R.E. Stratmann, O. Yazyev, A.J. Austin, R. Cammi, C. Pomelli, J.W. Ochterski, R.L. Martin, K. Morokuma, V.G. Zakrzewski, G.A. Voth, P. Salvador, J.J. Dannenberg, S. Dapprich, A.D. Daniels, O. Farkas, J.B. Foresman, J.V. Ortiz, J. Cioslowski, and D. J. Fox, Gaussian 09, Revision C.01, Gaussian, Inc., Wallingford CT, 2010;

4 Z. Zhao, H. Chen, B. Wu, L. Zhang, Z. Li, T. Feng and J. Liu, *J. Org. Chem.*, 2017, **82**, 7974-7979.

5 M. M. Zanardi, A. G. Suárez and A. M. Sarotti, *J. Org. Chem.*, 2017, **82**, 1873-1879.

6 N. Grimblat, M. M. Zanardi and A. M. Sarotti, *J. Org. Chem.*, 2015, **80**, 12526-12534.

7 S. G. Smith and J. M. Goodman, *J. Am. Chem. Soc.*, 2010, **132**, 12946-12959.

8 T. Bruhn, A. Schaumlöffel, Y. Hemberger and G. S. Bringmann, *SpecDis*, version 1.60; University of Würzburg: Würzburg, Germany, 2012.
